# Combined effect of BCG vaccination and enriched environment promote neurogenesis and spatial cognition via a shift in meningeal macrophage M2 polarization

**DOI:** 10.1186/s12974-017-0808-7

**Published:** 2017-02-10

**Authors:** Fangfang Qi, Zejie Zuo, Junhua Yang, Saisai Hu, Yang Yang, Qunfang Yuan, Juntao Zou, Kaihua Guo, Zhibin Yao

**Affiliations:** 1 Department of Anatomy and Neurobiology, Zhongshan School of Medicine, Sun Yat-sen University, #74, Zhongshan No.2 Road, 510080 Guangzhou, People’s Republic of China; 2 Guangdong Province Key Laboratory of Brain Function and Disease, Zhongshan School of Medicine, Sun Yat-sen University, #74, Zhongshan No. 2 Road, 510080 Guangzhou, People’s Republic of China

**Keywords:** Enriched environment, Vaccine, Th1/Th2 balance, Hippocampus, Neurotrophins, Cytokine

## Abstract

**Background:**

The spatial learning abilities of developing mice benefit from extrinsic cues, such as an enriched environment, with concomitant enhancement in cognitive functions. Interestingly, such enhancements can be further increased through intrinsic Bacillus Calmette-Guérin (BCG) vaccination.

**Results:**

Here, we first report that combined neonatal BCG vaccination and exposure to an enriched environment (Enr) induced combined neurobeneficial effects, including hippocampal long-term potentiation, and increased neurogenesis and spatial learning and memory, in mice exposed to the Enr and vaccinated with BCG relative to those in the Enr that did not receive BCG vaccination. Neonatal BCG vaccination markedly induced anti-inflammatory meningeal macrophage polarization both in regular and Enr breeding mice. The meninges are composed of the pia mater, dura mater, and choroid plexus. Alternatively, this anti-inflammatory activity of the meninges occurred simultaneously with increased expression of the neurotrophic factors BDNF/IGF-1 and the M2 microglial phenotype in the hippocampus. Our results reveal a critical role for BCG vaccination in the regulation of neurogenesis and spatial cognition through meningeal macrophage M2 polarization and neurotrophic factor expression; these effects were completely or partially prevented by minocycline or anti-IL-10 antibody treatment, respectively.

**Conclusions:**

Together, we first claim that immunological factor and environmental factor induce a combined effect on neurogenesis and cognition via a common pathway-meningeal macrophage M2 polarization. We also present a novel functional association between peripheral T lymphocytes and meningeal macrophages after evoking adaptive immune responses in the periphery whereby T lymphocytes are recruited to the meninges in response to systemic IFN-γ signaling. This leads to meningeal macrophage M2 polarization, subsequent to microglial M2 activation and neurotrophic factor expression, and eventually promotes a positive behavior.

**Electronic supplementary material:**

The online version of this article (doi:10.1186/s12974-017-0808-7) contains supplementary material, which is available to authorized users.

## Background

During early life when the brain is exquisitely sensitive to signals, both exogenous factors such as environmental and maternal care, as well as endogenous mediators, including immunological molecules and neurotrophins, affect brain development. An emerging body of literature has demonstrated that immune activation during the early postnatal period may markedly induce cognitive impairment after a subsequent immune challenge later in life [1]. Nevertheless, Bilbo et al. [2] also reported that neonatal immune activation caused by *Escherichia coli* protected against rather than aggravated stressor-induced depressive-like symptoms.

Another feasible scenario is that preconditioning with a low dose of lipopolysaccharide (LPS) can attenuate the pathological effects of a subsequent stimulus, such as a larger LPS challenge [3], brain trauma [4], or stress [5, 6]. Although the precise mechanism is not fully understood, facilitating M2 activation of resident microglia induced by LPS preconditioning at least partially explains this favorable phenomenon [7].

Interestingly, we recently reported that neonatal BCG vaccination modulates dendritic development, neurogenesis, and behavior in early life by inducing M2 microglial activation and a neurotrophic neuroimmune pattern in the brain [8–10]. In another study, we showed that infiltrating T lymphocytes in the choroid plexus (CP) contribute to induce M2 microglial polarization and secret neurotrophic factors during a normal physiological state [11]. In addition to T lymphocytes, monocyte-derived anti-inflammatory macrophages were also detected in the CP [12] and the pathologic central nervous system (CNS) [13]. This observation included CP activation after evoking the peripheral immune response without breakdown of the blood-brain barrier (BBB), where the myeloid cells exerts inflammation-resolving effects and thus fights off the CNS pathology. Importantly, mice trained in a spatial learning and memory test, the Morris water maze (MWM), led to recruitment of IL-4-producing T lymphocytes into the meninges, and depletion of T lymphocytes from the meningeal spaces skewed meningeal macrophages toward a pro-inflammatory phenotype [14].

Exposure to an enriched environment (Enr) has been suggested to exert positive effects on plasticity in the hippocampus and other regions in the brain, including elevated learning and memory, neurogenesis, cell survival [15], alterations in microglial phenotypes [16], antigen expression [17], and neuroimmune functions [18]. Enr increases the expression of growth factors, such as BDNF and IGF-1, in the brain. Moreover, peripheral T lymphocytes are essential for maintaining normal neurogenesis and cognition. Ziv et al. [17] also reported that an enriched environment did not enhance neurogenesis in immune deficient mice, whereas another form of cognitive training (MWM) indeed enhanced behavioral performance by an increasing meningeal T lymphocyte recruitment [14].

The hippocampal plasticity of newborns is more susceptible to environmental exposure than that of adults. Therefore, early enriched experience by pre-weaning into an Enr may induce pronounced effects on neurogenesis and behavior [19]. However, whether neonatal pretreatment with a BCG vaccination could further influence Enr-related elevated hippocampal neurogenesis and behavior and the precise mechanisms regulating this process remained elusive. Here, we show that BCG vaccination indeed further enhances hippocampal neurogenesis and spatial cognition caused by Enr by activating the CP, recruiting T lymphocytes to the CP, and increasing meningeal macrophage M2 polarization and neurotrophic factor expression.

## Methods

### Animals and housing conditions

All of the protocol were approved by the Animal Care and Use Committee of Sun Yat-sen University and conformed to the Guide for the Care and Use of Laboratory Animals by the National Institutes of Health, USA. Adult male and female C57BL/6 mice (8 weeks) were purchased from the Sun Yat-sen University Laboratory Animal Center (Guangzhou, China) and were housed in same-sex pairs in a specific pathogen-free facility. Several females and males were housed together so that multiple cohorts of newborn pups were available simultaneously. The colony was maintained on 12-h light/dark cycles with free access to food and water in a temperature- and humidity-controlled room.

The Enr paradigm for neonatal mice was based on a previous protocol with slight modifications [20]. In brief, the enriched rearing paradigm consisted of a large Plexiglas laboratory cage (50 cm × 50 cm × 30 cm), containing two running wheels, multiple plastic toys, small huts, ladders, and a set of tunnels. The objects were repositioned three times a week. The standard housing paradigm was a common cage measuring 25 cm × 15 cm × 15 cm. The Enr group was housed in groups of eight mice per cage in the enriched environment conditions, and the control group was housed in groups of four mice per cage in the regular environment conditions (Reg). At postnatal week 1 (PW1), littermates were housed in Reg or Enr conditions for 3 weeks (from PW1 to PW4). The bottoms of all Reg and Enr cages were lined with saw dust bedding.

#### Experimental procedures

Neonatal mice (postnatal week 0) from one dam received single subcutaneous injections of a freeze-dried living BCG suspension (D2-BP302 strain, Biological Institute of Shanghai, China) in a volume of 25 μl per animal containing approximately 10^5^ CFUs or equivalent sterile phosphate buffer solution (PBS), as previously described [8]. One or two dams with their pups (10 pups minimum) were transferred to Enr cages, whereas only one dam with its pups in Reg cages at PW1 remained for 3 weeks. The pups were weaned and separated from their mothers at postnatal day 21. A maximum of two pups per dam were used in all experiments to rule out maternal variables. Both the male and female pups were used in the present experiments according to a recent critical paper from NIH [21]. However, only male pups were applied to behavioral testing according to previous research [22].

#### Open-field test (OFT)

For the open-field exploration, mice were placed in a corner of a dimly lit chamber of the open-field arena at PW4, and exploration and rearing activities were monitored using the Flex-Field activity system (San Diego Instruments, CA). Mouse locomotor activity was traced and quantified using Flex-Field software and the number of beam breaks per 30 min as described previously [8].

#### Morris water maze (MWM)

The cognitive tests were completed using male offspring at PW4, as previously described in detail [17]. Briefly, during the acquisition phase, the mouse was given four training trials per day for five consecutive days to locate a stationary platform positioned 1.0 cm below the water surface in a pool, using the distal visual shape and object cues available in the testing room. The maximum time given for each trial was 60 s. One mouse failed to reach the platform within 60 s and was manually guided to the platform and remained there for 10 s before it was returned to its cage. The inter-trial interval for each mouse was 10 min. During the probe phase (day 6), the platform was removed, and mice were given a single trial lasting 60 s without available escape. During the reversal phase (days 7 and 8), the platform was placed in the quadrant opposite the original acquisition training, and the mouse was retrained for four training trials per day. Swimming trajectories were recorded using the MT-200 Morris water maze tracking system (MT-200, Chengdu, China). All behavioral tests were performed between 10:00 and 17:00 h in the light-off phase.

#### Administration of BrdU and tissue preparation

For cell proliferation analysis, six mice received three BrdU (Sigma-Aldrich, St. Louis, MO, USA) injections (50 mg/kg, i.p., once every 2 h) at PW3, then were deeply anesthetized with chloral hydrate (120 mg/kg, i.p.), and were perfused intracardially with 0.9% NaCl, followed by 4% paraformaldehyde (PFA) at 2 h after the third BrdU injection. For neuronal differentiation analysis, six mice received four BrdU injections (from PW3, one injection per every 12 h) and were sacrificed 7 or 21 days after the first BrdU injection. On day 7, the dentate gyri (DG) were analyzed for the number of BrdU^+^/DCX^+^, BrdU^+^/Iba-1^+^, and BrdU^+^/GFAP^+^ cells. On day 21, the newly mature neurons double labeled with BrdU and NeuN in the DG were determined. The brains were collected, post fixed overnight at 4 °C for 10 h and equilibrated in 30% sucrose solution. Then, 40-μm-thick frozen sections of the hippocampus were collected using a Leica SM2000R sliding microtome (Leica Microsystems, Richmond Hill, Ontario, Canada) for histological analysis.

#### Antibodies and reagents for immunofluorescence

The following primary antibodies were used: anti-BrdU monoclonal antibodies (1:400, Oxford Biotechnology, UK), goat anti-DCX (1:400; Santa Cruz Biotechnology, CA, USA), rabbit anti-BDNF (1:200; Abcam, Cambridge, MA, USA), goat anti-mouse IGF-1 (1:200; BD Bioscience, San Jose, CA, USA), rat anti-mouse anti-CD3 (eBioscience, Santa Clara, CA, USA), rabbit anti-Iba-1 (1:1000; Wako Chemical, Japan), mouse anti-GFAP (1:10,000; Sigma-Aldrich, St. Louis, MO, USA), mouse anti-IL-10 (1:200; BD Bioscience, San Jose, CA, USA), chicken anti-Arginase-1 (1:500; Merck Millipore, Germany), rat anti-CD11b (1:1000; BD Bioscience, San Jose, CA, USA), and mouse anti-NeuN (1:1000; Sigma-Aldrich, St. Louis, MO, USA). The following secondary antibodies were used: Alexa Fluor 594 donkey anti-rat, Alexa Fluor 488 donkey anti-goat, Alexa Fluor 555 goat anti-rabbit, Alexa Fluor 488 goat anti-rabbit, Alexa Fluor FITC goat anti-chicken, and Alexa Fluor 488 goat anti-mouse antibodies (1:400; Molecular Probes, Eugene, OR, USA). The detailed immunofluorescence protocols were previously described [23].

#### Confocal and MBF stereoinvestigation

Microscopic and quantitative analyses of BrdU^+^/DCX^+^, BrdU^+^/NeuN^+^, BrdU^+^/Iba-1^+^, and BrdU^+^/GFAP^+^ cells in the DG were completed using the optical-fractionator method with a stereology system stereo investigator (MicroBrightField, Inc., Williston, VT, USA) as previously described [23].

#### Electrophysiology

The stereotactic method and induction of hippocampal LTP were established according to previously described methods. Briefly, the mice were placed into a stereotactic device (Stoelting Instruments, Wood Dale, IL, USA), and their body temperatures were kept constant. Scalp incisions were made in the skull surface, and the bregma was exposed. A unipolar-recording electrode was located 2 mm posterior to the bregma and 1.4 mm lateral to the midline and deepened into the hilus of the DG; a bipolar-stimulating electrode was located ipsilaterally 2.5 mm lateral to lambda and deepened by 1.5 mm into the perforant pathway. Hippocampal LTP was induced by 400 Hz high-frequency stimulation (HFS) 20 min after baseline recordings in the DG in vivo. LTP is expressed as percentage potentiation of baseline (100%), and the significance is determined at 58–60 min after the HFS. Values from the final 2-min bin were compared between the immunized group and the corresponding control group using Student’s *t* tests.

#### Enzyme-linked immunosorbent assay (ELISA)

The concentration of IFN-γ, IL-4, TNF-a, IL-1β, and IL-6 both in blood serum and hippocampus were measured as previously described [23]. Growth factor BDNF and IGF-1 levels in the hippocampus were also assessed as previously described [23].

#### Real-time quantitative polymerase chain reaction (RT-PCR)

Total RNA was extracted separately from the meninges and hippocampi using TRIzol reagent (Sangon Biotech, Shanghai, China). Messenger (m)RNA (1 μg) was converted into cDNA using a GoScriptTMcDNA Reverse Transcription Kit (Promega, Madison, WI, USA). The expression of specific mRNAs was assayed using fluorescence-based real-time quantitative PCR (RT-PCR). Quantitative PCR reactions were performed using AceQ qPCR SYBR Green Master Mix (Vazyme Biotech Co., Ltd, Nanjing, China) in triplicate for each sample. β-Actin was chosen as the reference gene because of its stable expression in the meninges and hippocampi. The amplification cycles were 95 °C for 10 s and 60 °C for 30 s; the cycle number is 40, with the melt curve at 60–95 °C. At the end of the assay, a melting curve was constructed to evaluate the specificity of the reactions. All of the quantitative real-time PCR reactions were determined and analyzed using a Bio-Rad IQ5 Real-Time PCR System with the comparative Ct method and were normalized to β-actin. The list of primers used is presented in Additional file 1: Table S1.

### Statistical analysis

The results were analyzed by two-way ANOVA with the treatment (PBS and BCG) and environment (regular and enriched environments) as independent variables. Three-way ANOVA with repeated measures was used in Morris water maze test (treatment and environment and time), and when warranted, the LSD post hoc test was performed. Normality and variance homogeneity were verified using the Shapiro-Wilk test and Levene’s test, respectively. The data are presented as the means ± SEMs, and *p* values less than 0.05 were considered significant.

## Results

### Neonatal BCG vaccination enhances the effect of Enr on motor activity and spatial cognition

**Fig. 1:**
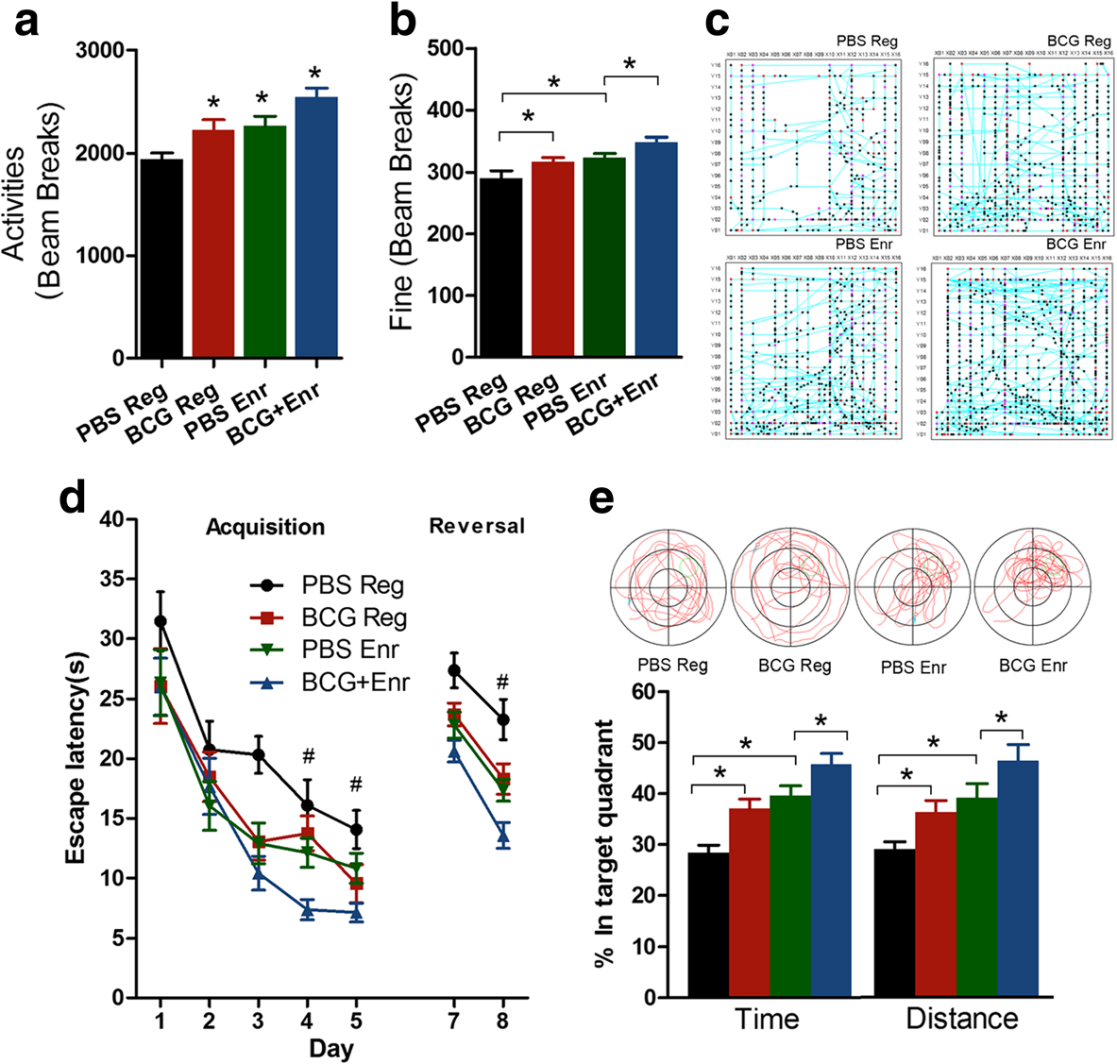
Neonatal BCG vaccination enhanced the effect of Enr on motor activity and spatial learning abilities. **a**, **b** Locomotor activities and fine activities (grooming) of BCG-Reg (*red*), PBS-Enr (*green*), BCG-Enr (*blue*), and the control mice (PBS-Reg; *black*). *p < 0.05, two-way ANOVA, LSD post hoc test. **c** Representative tracks of movement patterns of PBS-Reg, BCG-Reg, PBS-Enr, and BCG-Enr mice for 30 min. **d** Monitoring PBS-Reg, BCG-Reg, PBS-Enr, and BCG-Enr mice during the acquisition and reversal phases of a spatial cognitive task in the MWM test. ^#^ *p* < 0.05 three-way repeated-measures ANOVA, *n* = 10 per group. **e** Representative swimming trajectories of four groups in the extinction phase (*upper*). The time spent in the quadrant where the platform is located in the acquisition phase (*bottom*). *Asterisks* in **a** and **b** and *number sign* in **d** indicate the BCG Enr group compared with the PBS Enr group. *Asterisk* in **e** represents a significant difference between the indicated groups. The data are presented as the means ± SEMs

Significant effects were observed for the environment (motor activity: *F*_(1, 36)_ = 12.934, *p* < 0.001; fine: *F*_(1, 36)_ = 13.722, *p* < 0.001; *n* = 10) and vaccination (motor activity: *F*_(1, 36)_ = 10.113, *p* < 0.003; fine: *F*_(1, 36)_ = 8.946, *p* < 0.005, *n* = 10), but no interactions between the environment and vaccination (motor activity: *F*_(1, 36)_ = 0.000039, *p* > 0.05; fine: *F*_(1, 36)_ = 0.002, *p* > 0.05, *n* = 10) were observed regarding motor activity and fine activity (grooming). Neonatal mice vaccinated with BCG that were raised under regular conditions (BCG-Reg mice) showed more exploration and grooming than did their PBS-treated controls (PBS-Reg mice) (Fig. 1a, b). Similarly, there was also a significant increase for the BCG Enr mice (neonatal mice vaccinated with BCG that were raised in an enriched environment) toward displaying higher levels of exploration activity relative to the PBS-Enr mice (neonatal mice treated with PBS that were raised in an enriched environment) (Fig. 1a, b).

Our previous study demonstrated that early BCG vaccination also elevated spatial leaning abilities [8]. Therefore, cognitive testing was performed in the present animal model. The findings revealed that the BCG-Enr mice performed better than that did the PBS-Enr mice, as reflected by significant effects of environment and vaccination (environment: *F*_(1, 36)_ = 13.239, *p* < 0.001, *n* = 10; vaccination: *F*_(1, 36)_ = 10.103, *p* < 0.003, *n* = 10; interaction: *F*_(1, 36)_ = 1.387, *p* > 0.05), suggesting that the BCG-Enr mice displayed better spatial cognition than did the PBS-Enr mice (Fig. 1d). Briefly, during the acquisition of spatial learning, post hoc analyses indicated that the BCG-Enr mice were able to locate the hidden platform significantly faster with respect to both time and distance than the PBS-Enr vehicle mice (time: *p* < 0.05; distance: *p* < 0.05; Fig. 1e). Moreover, the BCG-Reg mice spent significantly less latent time and distance than did the PBS-Reg mice when locating the platform (time: *p* < 0.01; distance: *p* < 0.05; Fig. 1e), but these parameters were not statistically significantly different between the PBS-Reg mice and Enr-Reg mice (*p* > 0.05; Fig. 1e). There was a trend toward differences in swim speed between the Enr mice and the Reg mice, but these differences were not significant (data not shown).

### Neonatal BCG vaccination enhances the effect of Enr on hippocampal neurogenesis but does not affect neuroglia differentiation

**Fig. 2:**
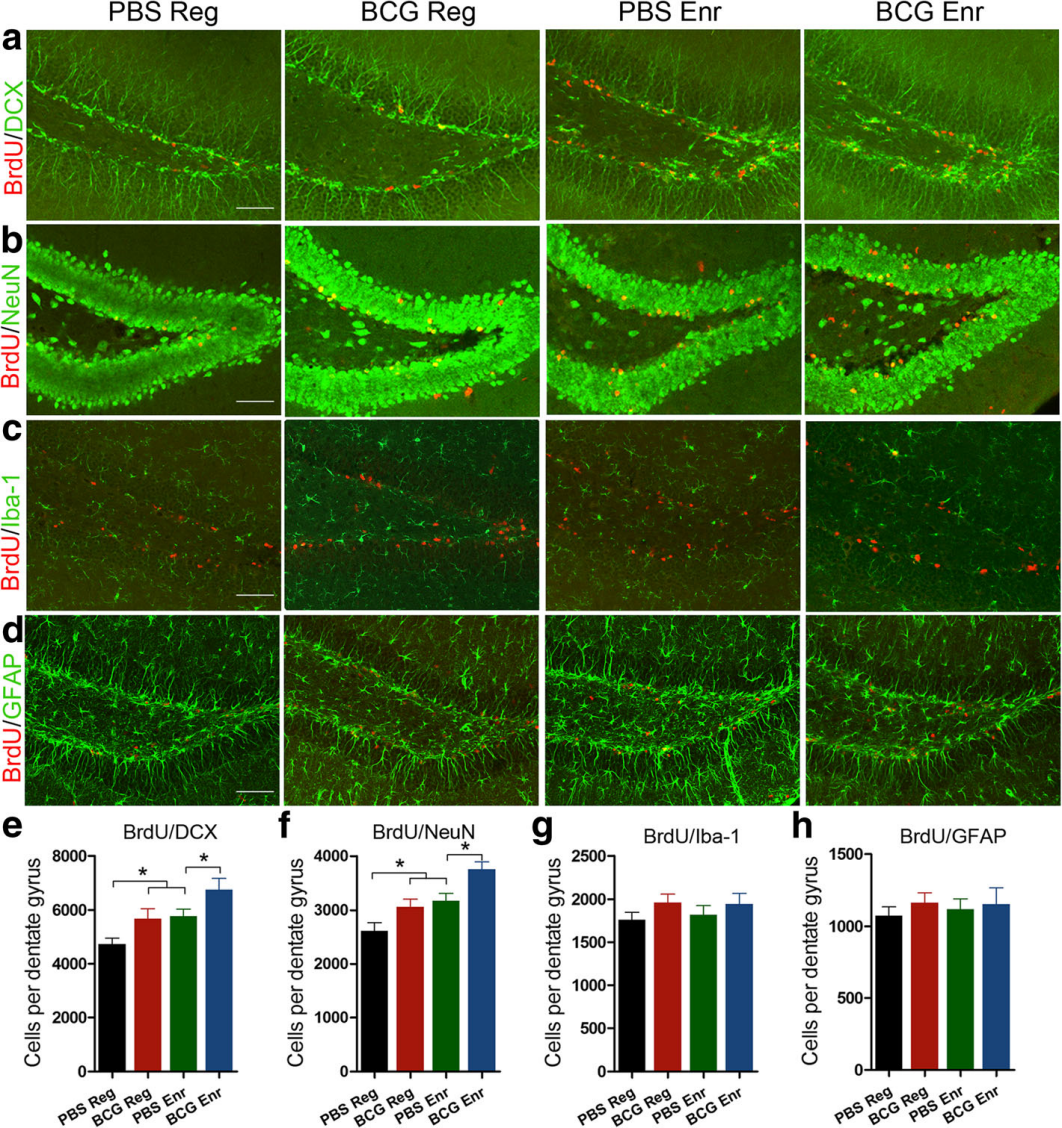
Neonatal BCG vaccination enhanced the effect of Enr on hippocampal neurogenesis but does not affect neuroglia differentiation. **a**–**d** Representative micrographs of the DG stained for BrdU (*red*) and with DCX staining (**a**, *green*), BrdU/NeuN (**b**), BrdU/Iba-1 (**c**), and BrdU/GFAP (**d**) from the PBS-Reg, BCG-Reg, PBS-Enr, and BCG-Enr mice. **e–h** Quantification of BrdU^+^/DCX^+^ cells (**e**), BrdU^+^/NeuN^+^ cells (**f**), BrdU^+^/Iba-1^+^ cells (**g**), and BrdU^+^/GFAP^+^ cells in the DG among all of the groups. *p < 0.05, two-way ANOVA, followed by LSD post hoc test; *n* = 6 per group. *Scale bars* 50 μm. The data are presented as the means ± SEMs

Spatial learning abilities are closely associated with hippocampal neurogenesis in the dentate gyrus (DG) [24, 25]. We thus assessed whether this correlation applies to the BCG-Enr mice that exhibited better hippocampal-dependent learning and memory than did the PBS-Enr mice. The main effect of Enr on hippocampal neurogenesis, including on BrdU^+^, BrdU^+^/DCX^+^, and BrdU^+^/NeuN^+^ cells, was measured (Enr, *F*_(1, 20)_ = 10.758, *p* < 0.005; *F*_(1, 20)_ = 10.797, *p* < 0.005; *F*_(1, 20)_ = 18.994, *p* < 0.001; *n* = 6, respectively), and significantly more BrdU^+^, BrdU^+^/DCX^+^, and BrdU^+^/NeuN^+^ cells were observed in the DG of the BCG-Enr mice than in that of their PBS-Enr counterparts, as reflected by the significant effect (Additional file 2: Figure S2, Fig. 2a, b; vaccination, *F*_(1, 20)_ = 17.526, *p* < 0.0001; *F*_(1, 20)_ = 8.986, *p* < 0.01; *F*_(1, 20)_ = 12.763, *p* < 0.002, *n* = 6 respectively). However, the number of BrdU^+^/Iba-1^+^ (Fig. 2c; Enr, *F*_(1, 20)_ = 0.040, *p* > 0.1; vaccination, *F*_(1, 20)_ = 2.463, *p* > 0.1, *n* = 6) and BrdU^+^/GFAP^+^ (Fig. 2d; Enr, *F*_(1, 20)_ = 0.037, *p* > 0.1; vaccination, *F*_(1, 20)_ = 0.593, *p* > 0.1, *n* = 6) cells in the DG of the BCG-Enr mice did not differ from than that of the PBS-Enr mice. These findings imply that neonatal BCG vaccination increases Enr-induced enhancement of hippocampal neurogenesis but not affect neuroglia differentiation.

### Hippocampal long-term potentiation (LTP) partially explains the enhanced cognitive function induced by BCG vaccination

**Fig. 3:**
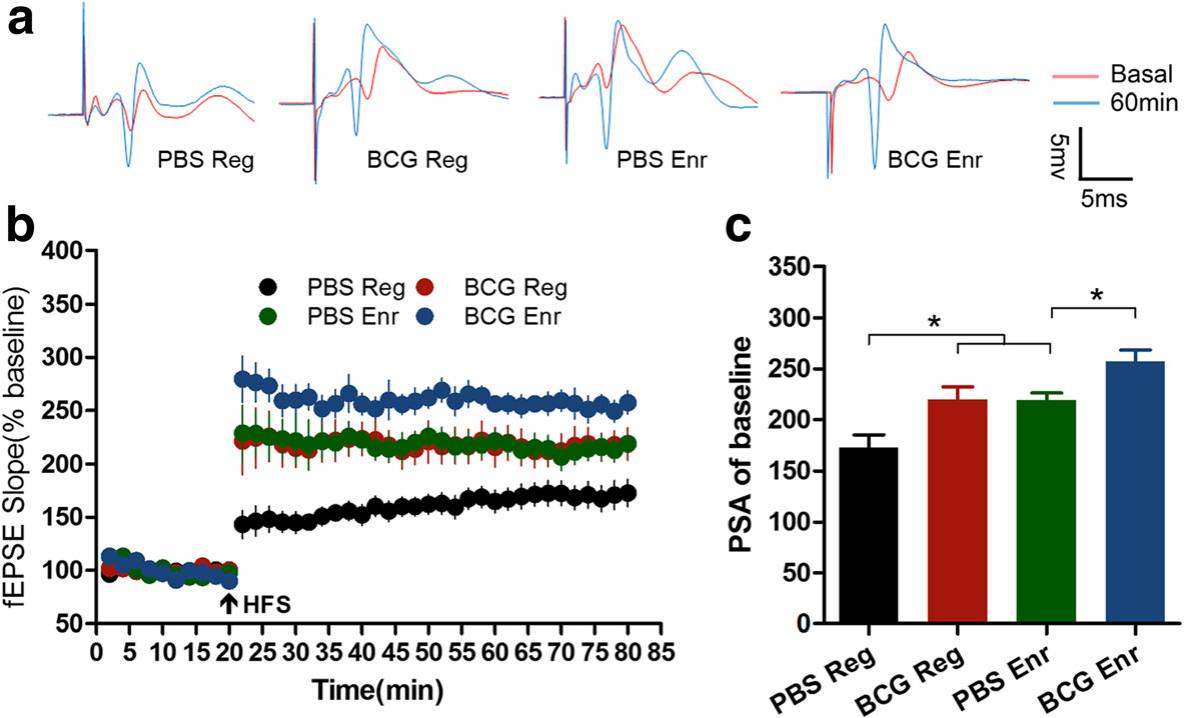
BCG vaccination induced hippocampal long-term potentiation induced by high-frequency stimulation (HFS) 20 min after baseline recordings in the DG in vivo. LTP is expressed as percentage potentiation of baseline (100%), and the significance is determined at 58–60 min after the HFS was given. **a** Traces (*top*) represent a typical response before (*red line*) and after (*blue line*) HFS. **b** BCG vaccination induces higher LTP both in mice raised both in regular environment and an enriched environment. **c** Potentiation of fEPSP at 58–60 min after HFS. Data in **c** are presented as the means ± SEMs (*p < 0.05; two-way ANOVA, LSD post hoc test, *n* = 6–8 per group)

Hippocampal LTP models the changes in synaptic plasticity that is thought to participate in spatial learning abilities and neurogenesis [26]. To verify the mechanism responsible for the cognitive function enhancement caused by BCG vaccination, we measured LTP from the medial perforant path to the dentate gyrus synapses to evaluate whether immune responses affect cognitive function via modulation of electrophysiological properties. This approach revealed that hippocampal LTP induced by high-frequency stimulus (HFS) was significantly increased in the BCG-Enr mice versus those of the mice in Enr housing, consistent with our observations at the behavioral and cellular level (Fig. 3; Enr: *F*_(1, 23)_ = 11.772, *p* < 0.01; vaccination: *F*_(1, 23)_ = 11.554, *p* < 0.01; interaction: *F*_(1, 23)_ = 0.087, *p* > 0.1; BCG-Enr vs PBS-Enr: *p* < 0.05, post hoc test; *n* = 6–8). These results indicate that BCG vaccination causes enhanced hippocampal LTP, and those alterations are responsible for learning and memory elevation in mice following Enr exposure.

### Neonatal BCG vaccination induces similar cytokine profiles between the hippocampus and the periphery

**Fig. 4:**
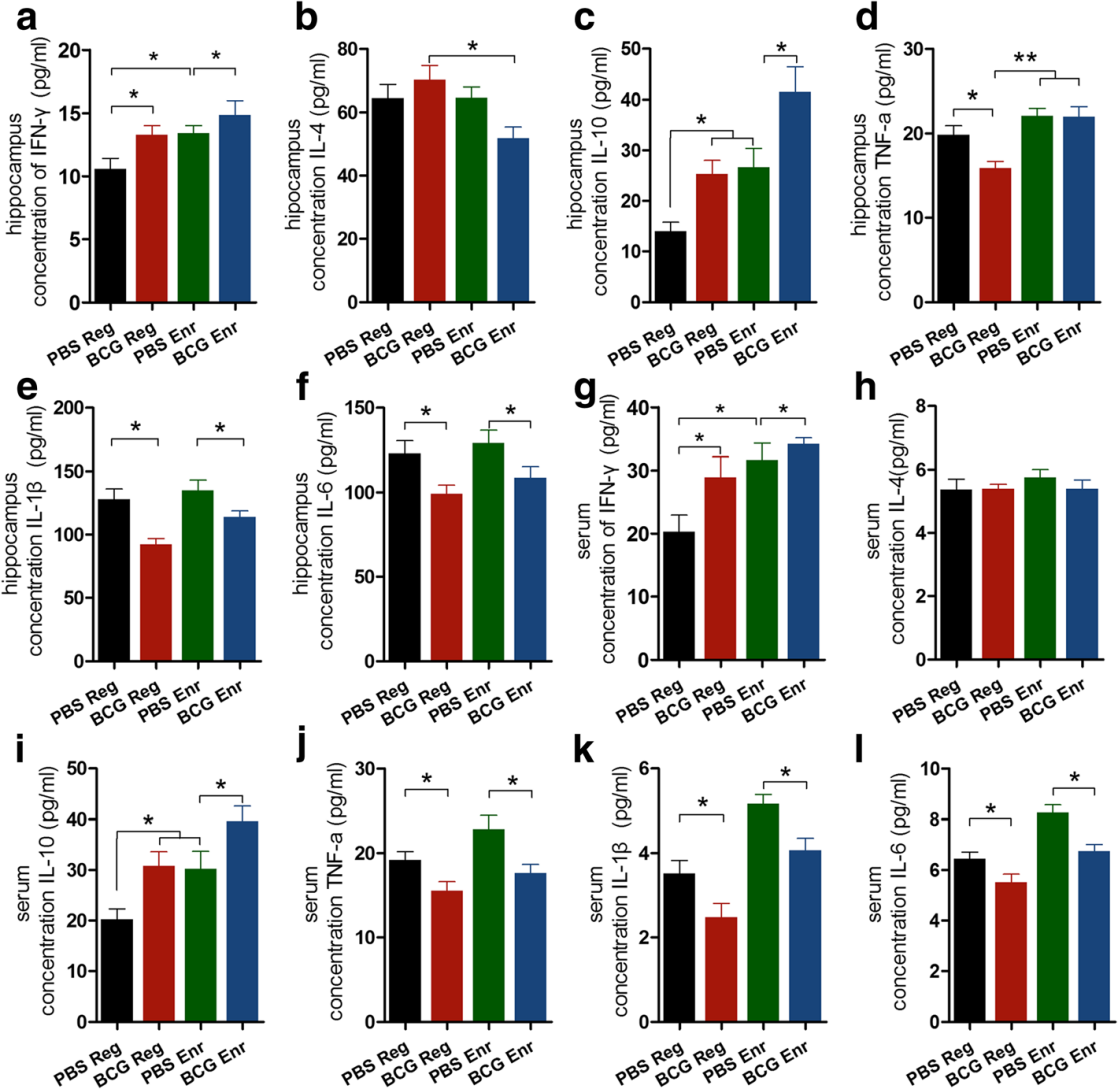
Effect of BCG vaccination on the expression of cytokines in the serum and hippocampus. The levels of IFN-γ, IL-4, IL-10, TNF-a, IL-1β, and IL-6 in the hippocampus (**a**–**f**) and serum (**g**–**l**) were quantitatively analyzed by ELISA. *p < 0.05, **p < 0.01; two-way ANOVA, LSD post hoc test; *n* = 6 per group. The data are presented as the means ± SEMs

**Fig. 5:**
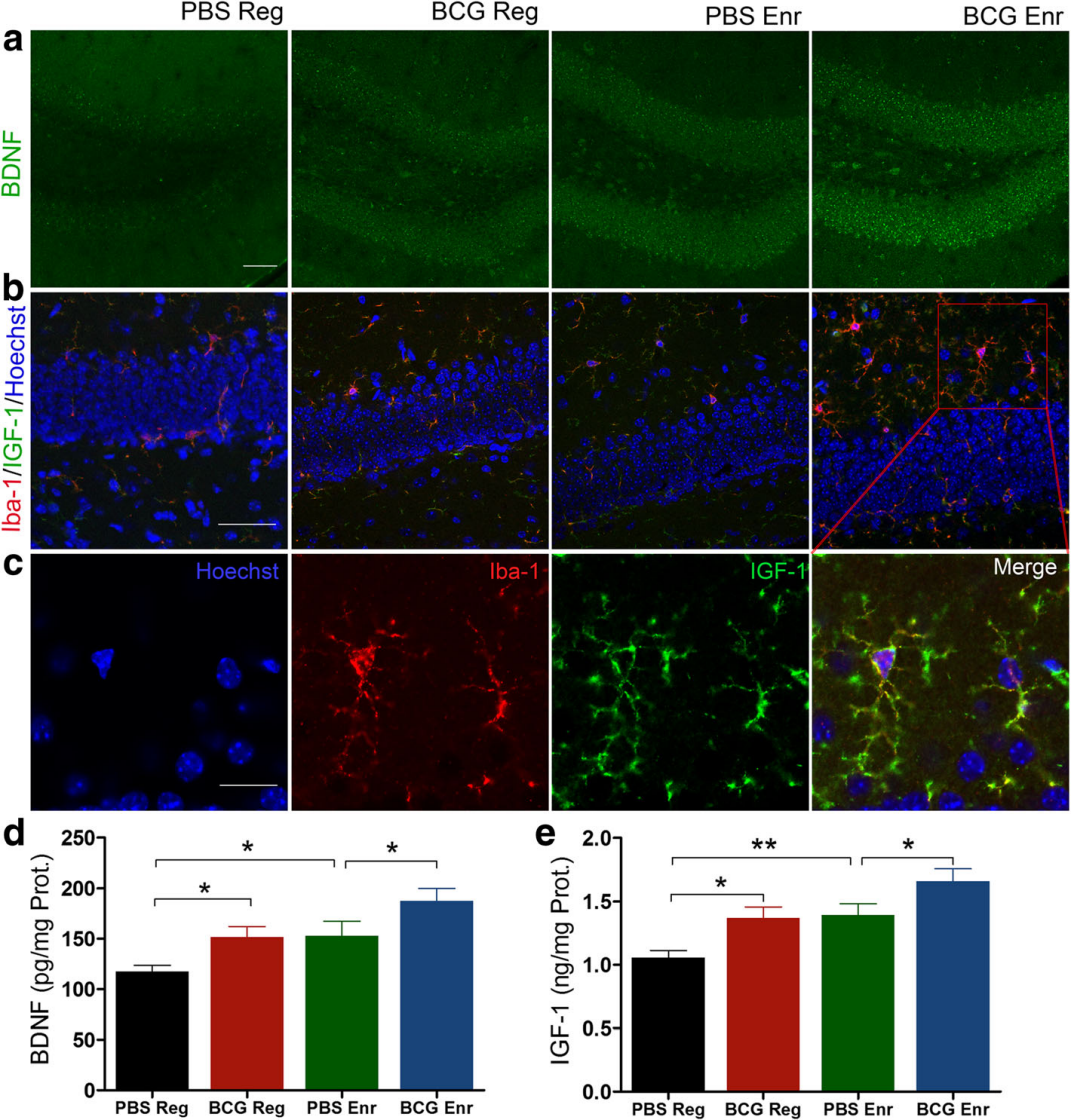
BCG vaccination elevated BDNF and IGF-1 levels in the hippocampus. **a** Confocal micrographs in the DG stained for BDNF. **b** Confocal micrographs in the DG stained for Iba-1 (*red*) and IGF-1 (*green*). **c** A higher magnification and single channels of the *inset* boxed area in **b. d**, **e** Quantification of BDNF and IGF-1 levels in the hippocampi of the four groups. *Scale bars* 50 μm in **a** and **b**; 20 μm in **c**; *p < 0.05, **p < 0.01 between indicated groups by two-way ANOVA, followed by LSD post hoc test. The data are presented as the means ± SEMs

Hippocampal neurogenesis is maintained by a pool of neural stem cells, which are located in a specialized microenvironment identified as a “neurogenic niche.” Next, we determined whether there are changes in secreted molecules, such as cytokines or growth factors, in the hippocampus as a result of neonatal immune activation that can potentially account for the enhanced neuronal plasticity of the BCG-Enr mice. The results revealed significantly increased levels of IFN-γ and reduced levels of IL-1β and IL-6 in the hippocampus and serum of the BCG-Enr mice. These findings were reflected by the significant effect of BCG vaccination on cytokines (Fig. 4a, e, f; hippocampus: IFN-γ, *F*_(1, 16)_ = 5.843, *p* < 0.05; IL-1β, *F*_(1, 20)_ = 18.702, *p* < 0.001; IL-6, *F*_(1, 20)_ = 10.840, *p* < 0.01; Fig. 4g, k, l; serum: IFN-γ, *F*_(1, 22)_ = 4.766, *p* < 0.05; IL-1β, *F*_(1, 20)_ = 13.975, *p* < 0.01; IL-6, *F*_(1, 20)_ = 17.756, *p* < 0.001; *n* = 5-8, two-way ANOVA) with no interactions observed between vaccination and Enr (*p* > 0.05 for all of the tests). In particular, we found that the neurotrophic factors BDNF and IGF-1 were significantly upregulated in the BCG-Enr mice compared with expression in the Enr mice, which is in line with reports that BDNF and IGF-1 are necessary for neurogenesis and synaptic transmission in the hippocampus (Fig. 5a–e; BDNF: vaccination, *F*_(1, 20)_ = 9.258, *p* < 0.01; Enr, *F*_(1, 20)_ = 9.892, *p* < 0.01; IGF-1: vaccination, *F*_(1, 20)_ = 11.924, *p* < 0.003; Enr, *F*_(1, 20)_ = 14.068, *p* < 0.001; *n* = 6). These data suggest neonatal BCG vaccination triggers adaptive peripheral immune responses and thereby regulates central cytokines and NT secretion to build a favorable neurogenic niche and ultimately modulate hippocampal neurogenesis and LTP.

### BCG vaccination induces meningeal macrophage M2 polarization; this effect could be prevented by minocycline treatment

**Fig. 6:**
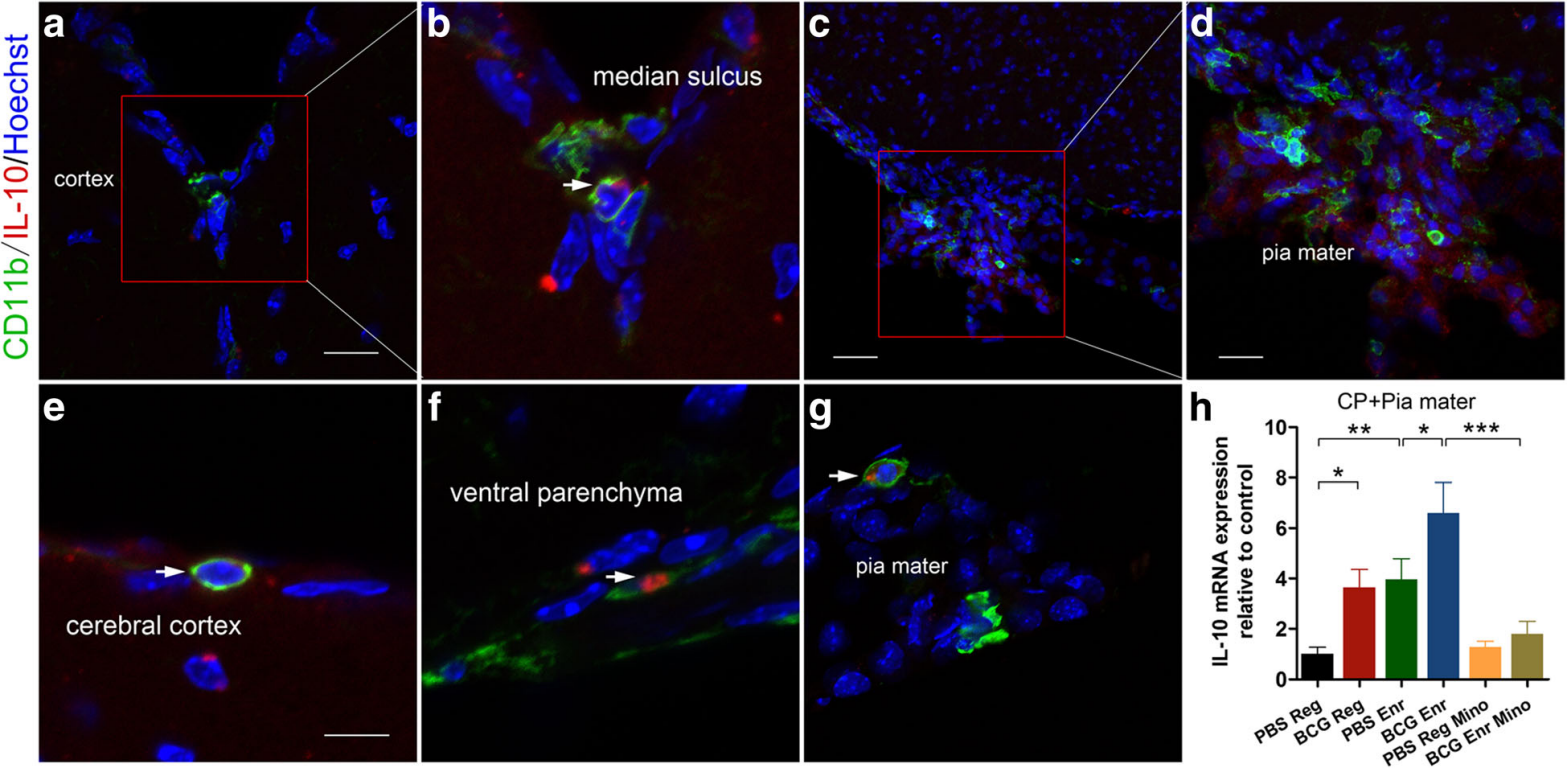
BCG vaccination induced pia mater macrophage secretion of anti-inflammatory cytokine IL-10; the effect could be prevented by minocycline treatment. **a** Representative micrographs of the median sulcus of cortex stained for CD11b (*green*) and IL-10 (*red*) from the BCG Enr group. **b** A higher magnification (×63 and zoom 2.5) of the *inset* boxed area in **a. c**, **e**, **f** Confocal micrographs of ventral (**c**, **f**) and dorsal parenchyma (**e**) of the cortex stained for CD11b (*green*) and IL-10 (*red*) from the BCG Enr group. **d** A higher magnification of the *inset* boxed area in **c. g** Confocal micrographs of pia mater stained for CD11b and IL-10 from BCG Enr group. **h** Quantification of IL-10 mRNA levels of different groups. *p < 0.05, **p < 0.01, ***p < 0.001; Student’s *t* test for comparison of BCG Enr group and BCG Enr-treated minocycline group, and two-way ANOVA, LSD post hoc test for others; *n* = 6 per group. *Scale bars* 20 μm in **a**; 100 μm in **c**; 50 μm in **d**; 10 μm in **b**, **e**, **f**, and **g**. The data are presented as the means ± SEMs

A fundamental question arising from these findings is how the peripheral immune response and central neurogenic niche interact and thereby regulate the central nervous system (CNS). Thus, we decided to assess the mechanisms by which macrophages function in the pia mater as a prerequisite for the influence of these cells on cognitive function [27]. The data revealed that meningeal macrophages expressed more ample anti-inflammatory IL-10 in the BCG-Enr mice relative to the PBS-Enr mice, indicating that BCG vaccination shewed macrophages toward an M2 anti-inflammatory phenotype (Fig. 6h; vaccination: *F*_(1, 12)_ = 9.962, *p* < 0.01; Enr: *F*_(1, 12)_ = 12.571, *p* < 0.01; *n* = 4; two-way ANOVA). Interestingly, meningeal macrophages remained in a quiescent state and expressed small amounts of IL-10 in the PBS-Reg mice.

Minocycline, a semisynthetic tetracycline derivative, is known to inhibit myeloid cell activation in vivo [11, 28]. To inhibit sustained macrophage activation after BCG vaccination, we injected minocycline (20 mg/kg; Sigma-Aldrich) intraperitoneally into the PBS-Enr mice and the BCG-Enr mice. We confirmed that BCG-induced macrophage activation (M2 phenotype) was inhibited by minocycline treatment (Fig. 6h; *p* < 0.01, post hoc test).

**Fig. 7:**
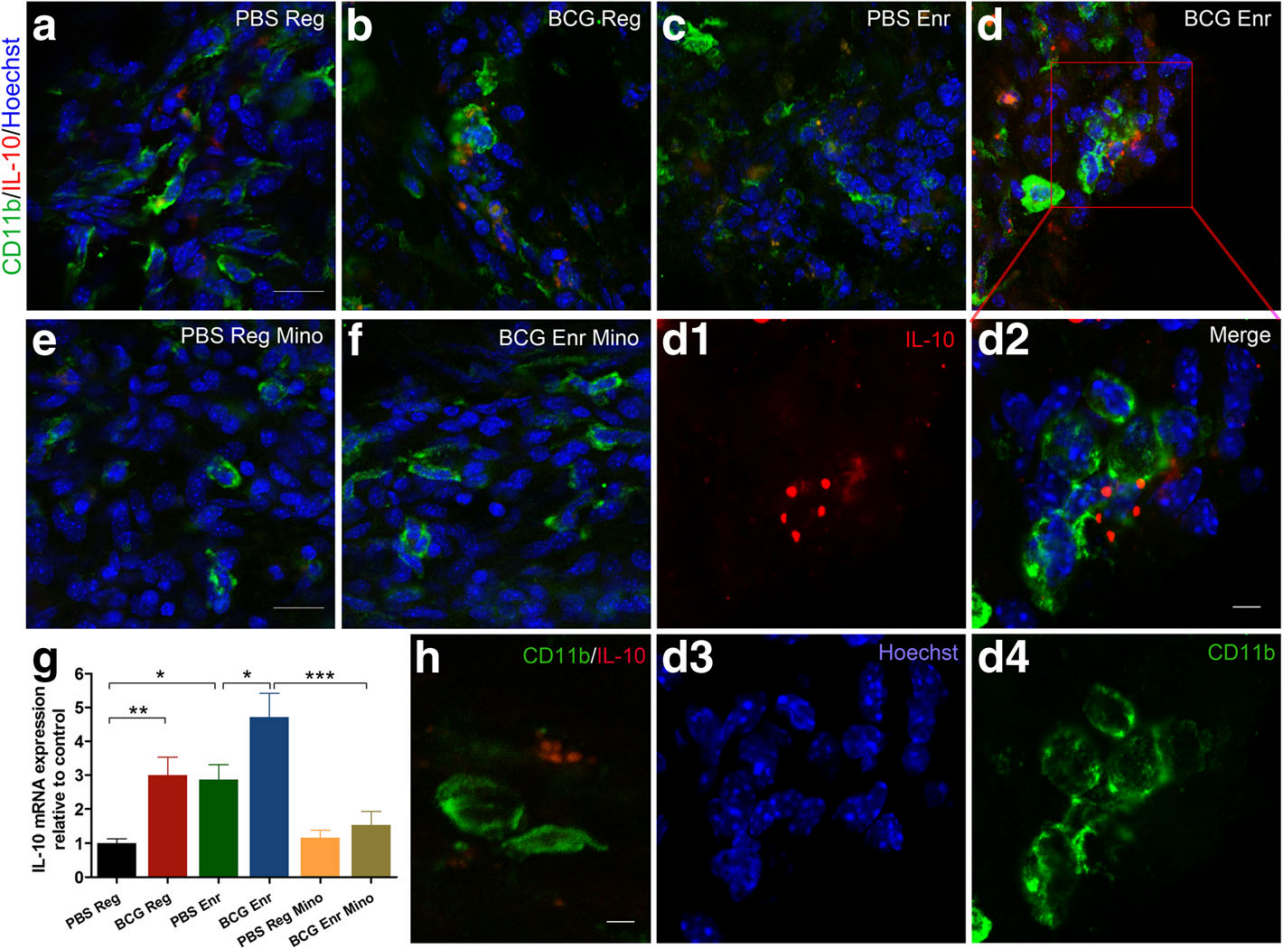
BCG vaccination induced dura mater macrophage secretion of anti-inflammatory cytokine IL-10; this effect could be prevented by minocycline treatment. **a**–**f** Representative micrographs of the dura mater stained for CD11b (*green*) and IL-10 (*red*) from the PBS-Reg group (**a**), BCG-Reg group (**b**), PBS-Enr group (**c**), BCG-Enr group (**d**), PBS-Reg group treated with Mino (**e**), and BCG-Enr group treated with Mino (**f**). **d1**–**d4** A higher magnification and single channels of the *inset* boxed area in **d. g** Analysis of IL-10 mRNA levels of the dura mater among different groups. **h** Immunostaining for IL-10 (*red*) and macrophages (CD11b^+^, *green*) of the dura mater. *p < 0.05, **p < 0.01, ***p < 0.001; Student’s *t* test for comparison of BCG-Enr groups treated with minocycline or not, and two-way ANOVA, LSD post hoc test for others; *n* = 6 per group. *Scale bars* 20 μm in **a**–**f**; 5 μm in **d1**–**d4** and in **h**. The data are presented as the means ± SEMs

Considering one side of pia mater contacts the cerebrospinal fluid (CSF) and the other side warps the brain due to its anatomical structure [29], we examined the macrophage-derived anti-inflammatory IL-10 in the pia mater. Obviously elevated immunostaining for IL-10 was observed in the ventral and dorsal pia mater in the BCG-Enr mice, whereas these effects were prevented by minocycline treatment (Fig. 6e, f). As an important component of the meninges, the dura mater contains lymphatics and immune cells that contribute to enable the drainage of macromolecules. Representative fluorescence micrographs revealed low levels of IL-10 expression in the dura mater of the PBS-Reg mice, whereas obvious increases in IL-10 levels were observed, especially as expressed by CD11b^+^ rounded macrophages in the dura mater of the BCG-Enr mice (Fig. 7a–d; vaccination: *F*_(1, 12)_ = 14.774, *p* < 0.001; Enr: *F*_(1, 12)_ = 12.852, *p* < 0.01; *n* = 6; two-way ANOVA). Similarly, low levels of IL-10 expression were found in the dura mater of the BCG-Enr mice treated with minocycline (Fig. 7f; *p* < 0.01, post hoc test). To further identify M2 macrophages in the meninges and brain tissue, we detected M2 genes (Arginase-1 and Ym1) in hippocampi from the four groups. The BCG-Reg mice showed increased basal expression of the M2 gene Arg-1 and Ym1 relative to that of the PBS-Reg mice (Additional file 3: Figure S4H and I; *p* < 0.05 for Arg-1; *p* < 0.001 for Ym1, post hoc test). Expectedly, compared with the PBS-Enr mice, the expression of the Arg-1 gene was significantly increased in the BCG-Enr mice (Additional file 3: Figure S4H and I; Arg-1; vaccination: *F*_(1, 8)_ = 13.299, *p* < 0.01; Enr: *F*_(1, 8)_ = 25.195, *p* < 0.001; Ym1; vaccination: *F*_(1, 8)_ = 2.241, *p* > 0.1; Enr: *F*_(1, 8)_ = 4.385, *p* < 0.07). Interestingly, the elevated M2-gene levels were normalized in the BCG-Enr mice following minocycline treatment (data not shown). Immunofluorescence analysis also confirmed obvious increase in Arg-1 expression in the meninges and brain tissue, including hippocampus, lateral, and third ventricles of the BCG-Enr mice (Additional file 3: Figure S4A-G).

Accordingly, it appears that more macrophage M2 polarization in the meninges correlates with diminished levels of IL-1β and IL-6 and increased levels of BDNF and IGF-1 in the hippocampus (data not shown). Thus, these results suggested that meningeal (pia mater and dura mater) macrophage polarization by secretion of anti-inflammatory IL-10 serves as a neuroimmune interaction between the peripheral immune response caused by BCG immunization and cognitive function.

### Recruitment of T lymphocytes to the choroid plexus (CP) is a requirement for meningeal macrophage activation

**Fig. 8:**
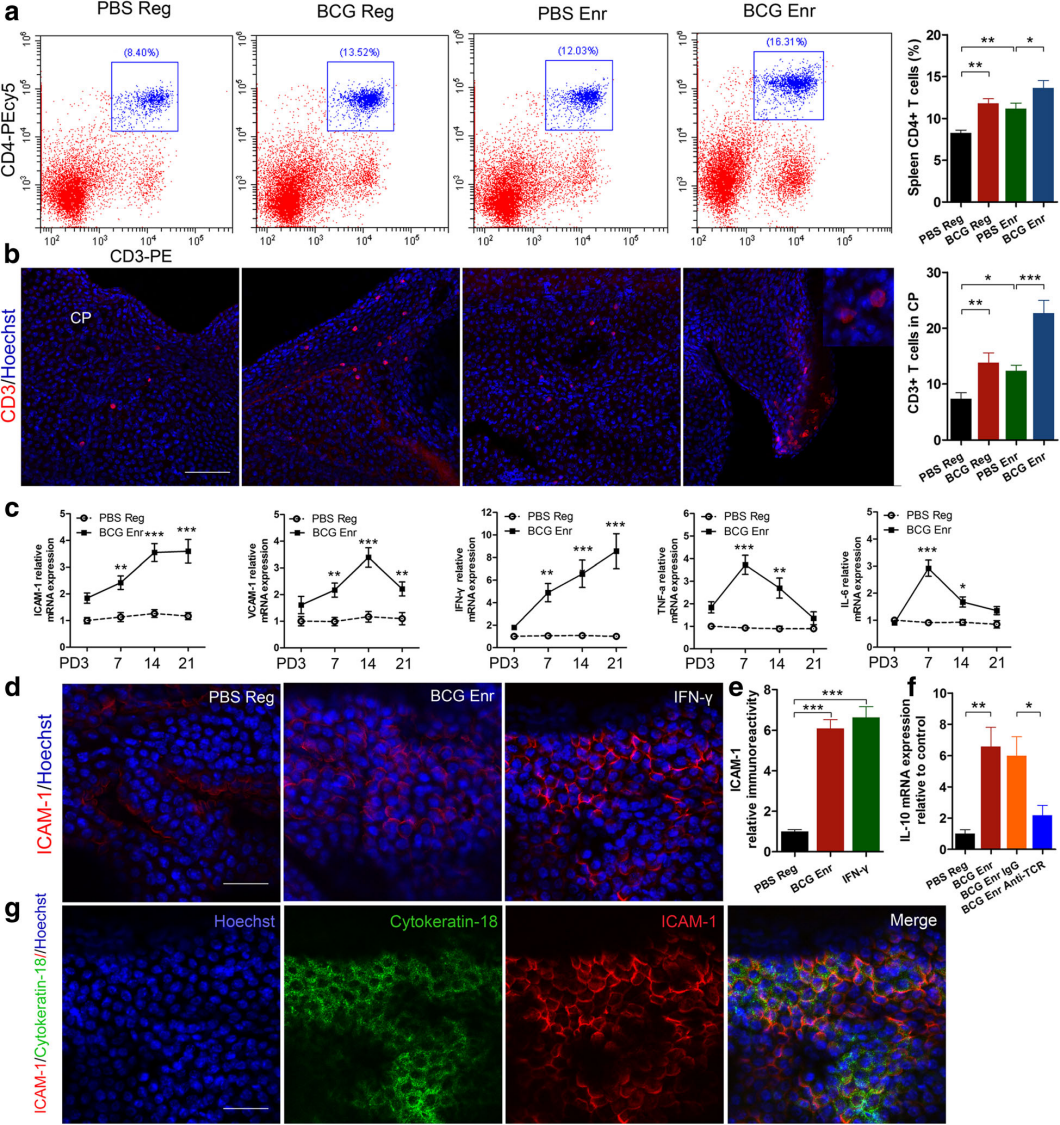
Recruitment of T lymphocytes into the choroid plexus (*CP*) is a requirement for the meningeal macrophage activation. **a**, **b** Quantitative analyses of the number of CD4^+^ T cells in the spleen (**a**) and CPs (**b**) of the PBS-Reg group, BCG-Reg group, PBS-Enr group, and the BCG-Enr group. **c** The graphs show expression analyses of the ICAM-1 gene, VCAM-1 gene, IFN-γ gene, TNF-a gene, and IL-6 gene in the CP of the BCG-Enr mice relative to the controls (PBS-Reg mice) at the indicated times (PD3, PD7, PD14, and PD21). **d**, **e** Representative micrographs (**d**) and analyses (**e**) of the CPs stained for ICAM-1 (*red*) and with nuclear staining for Hoechst (*blue*) from the PBS-Reg group, BCG-Enr group, and the PBS-Reg group treated with IFN-γ. **f** Quantitative analyses by RT-PCR of IL-10 mRNA levels in the CPs from PBS Reg group, BCG Reg group, and PBS Enr group treated with IgG or anti-TCR antibodies. **g** Confocal micrographs of the CPs stained for ICAM-1 (*red*) and cytokeratin-18 (*green*) from PBS Reg treated with IFN-γ group. *p < 0.05, **p < 0.01, ***p < 0.001 between the indicated groups by two-way ANOVA, LSD post hoc test, and Student’s *t* test (*n* = 6 per group). *Scale bars* 100 μm in **b**; 50 μm in **d** and in **g**. The data are presented as the means ± SEMs

The above findings promoted us to explore how peripheral BCG vaccination induced immune response affects meningeal macrophage activation. A recent report indicated that T lymphocytes infiltrating the CP or meninges appear to play a role in homeostatic regulation of cognitive function via meningeal myeloid cell immunity [14]. To address this interaction between T lymphocytes and macrophage activation, we stained for T lymphocytes in the whole-mount CP, which could be representative of the observations made in the meninges using immunofluorescence method. At 21 days after the vaccination, we found a significant elevation of T lymphocytes in the spleen and in the CP of BCG-Enr mice compared with those in PBS-Enr mice (Fig. 8a, b; CP, vaccination: *F*_(1, 20)_ = 26.831, *p* < 0.001; Enr: *F*_(1, 20)_ = 18.119, *p* < 0.001; spleen, vaccination: *F*_(1, 20)_ = 21.809, *p* < 0.001; Enr: *F*_(1, 20)_ = 13.453, *p* < 0.01; *n* = 6; two-way ANOVA). The increased recruitment of T lymphocytes to the CP paralleled observed increases in macrophage M2 polarization in the meningeal spaces (Additional file 4: Figure S1; dura mater: *r* ^2^ = 0.254, *p* < 0.05; pia mater: *r* ^2^ = 0.510, *p* < 0.002). The results are reminiscent of a recent report in which depletion of T lymphocytes from the meningeal spaces skewed meningeal macrophages toward a proinflammatory phenotype [14]. Then, to determine whether T lymphocytes infiltrating the CP are prerequisite for meningeal macrophage activation in response to BCG vaccination, we showed that depletion of T lymphocytes in the CP by treatment with anti-TCR antibodies abolished the meningeal macrophage M2 polarization (Fig. 8f; BCG-Enr anti-TCR group vs BCG-Enr IgG group, *p* < 0.05, Student’s *t* test). In addition, we also found a significant increase of T lymphocytes in the dura mater of the BCG-Enr mice compared with those of the PBS-Enr mice; similar results were observed between the BCG-Reg mice and the PBS-Reg mice (Additional file 5: Figure S3; vaccination: *F*_(1, 20)_ = 77.430, *p* < 0.0001; Enr: *F*_(1, 8)_ = 41.364, *p* < 0.0001; *n* = 6; two-way ANOVA).

### IFN-γ-dependent activation of the choroid plexus and the dura mater supports the recruitment of lymphocytes in response to BCG vaccination and/or enriched environment exposure

The CP and dura mater of BCG-Reg and/or Enr mice are populated by T lymphocytes, and T cell-derived IFN-γ was founded to be necessary for inducing expression of adhesion molecules by the CP epithelium by us [11] and others [30]. To further support this hypothesis in our animal model, we measured the expression levels of cytokines and trafficking molecules in the different groups. We found significant permanent increases in the levels of IFN-γ, ICAM-1, and VCAM-1 mRNA expression in the BCG-Enr mice compared with the PBS-Reg mice, whereas there were dual alterations in the TNF-a and IL-6 mRNA levels (Fig. 8c). To determine whether the cytokine IFN-γ was relevant to the induction of adhesion molecule expression following the BCG vaccination and/or exposure to the Enr, we infused the recombinant cytokine IFN-γ or vehicle to the unilateral lateral ventricle using a subcutaneous osmotic minipump (rate of infusion 1 μl/h) for 7 days as previously described. The recombinant IFN-γ treatment indeed increased the level of adhesion-related molecule, such as ICAM-1, reaching a level comparable to that observed in the BCG-Enr mice (Fig. 8d, e; BCG-Enr mice vs IFN-γ treated mice, *p* > 0.05, student’s *t* test). To determine the role of IFN-γ in more detail, we analyzed the infiltrating T cells in the dura mater and the levels of chemokines after intravenous injection of recombinant IFN-γ. The results revealed that IFN-γ induced the expression of CCL5, which may explain why much more T cells were recruited from periphery after IFN-γ injection (Additional file 6: Figure S5E and F). Together, the data further supported our hypothesis that increased adhesion molecules and chemokines contributed to the recruitment of peripheral T lymphocytes to the CP and dura mater in an IFN-γ-dependent manner in response to BCG vaccination and/or enriched environment housing.

### The essential role of meningeal macrophage activation in neurotrophic factor expression

**Fig. 9:**
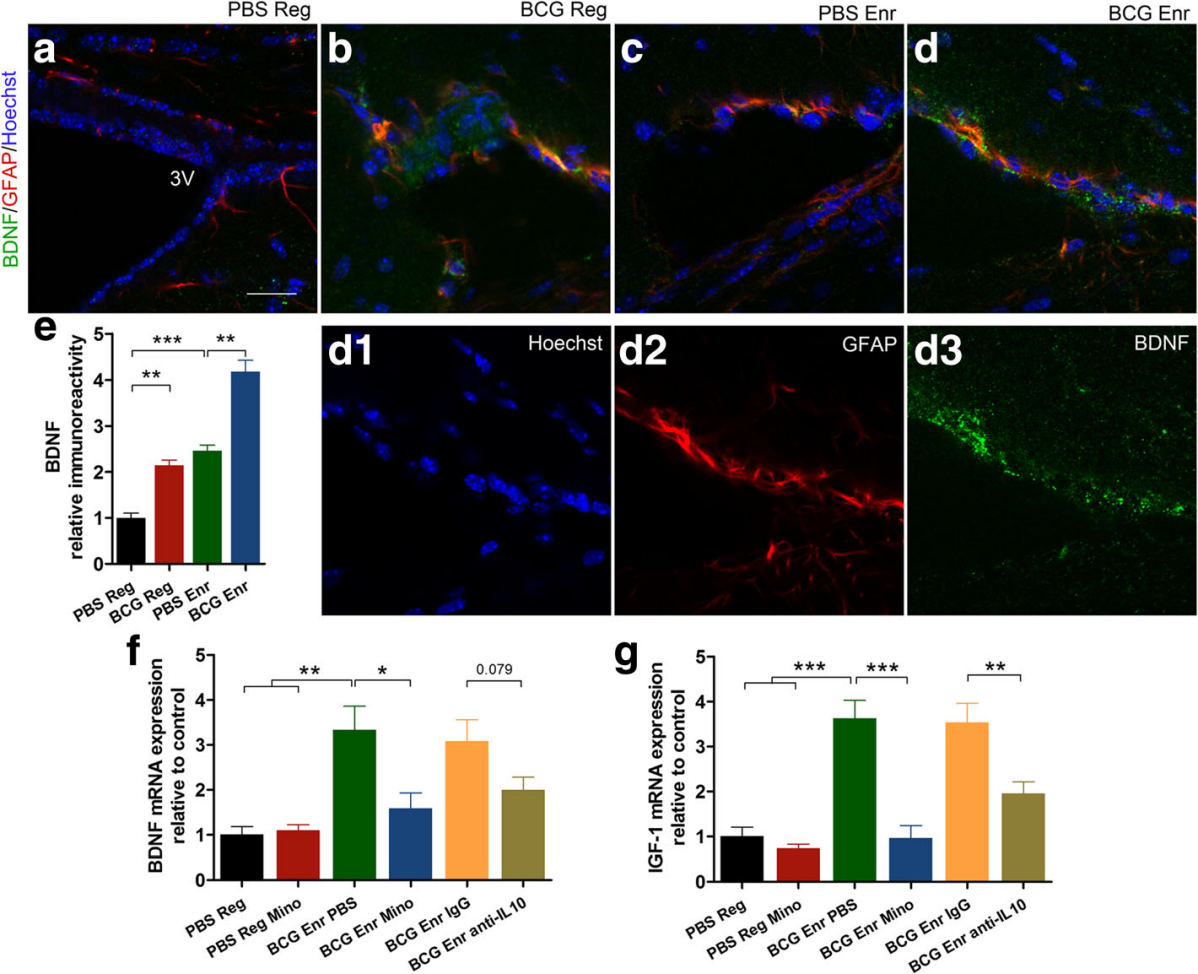
BCG vaccination enhances expression of hippocampal BDNF and IGF-1; this effect could be partially prevented by minocycline and anti-IL-10 antibody treatment (60 μg/mouse, BD Bioscience). **a**–**d** Representative confocal images of astrocytes (GFAP^+^) lining brain borders secreting BDNF (*green*), 3V, third ventricle. **d1**–**d3** Single channels of image (**d**) are presented. **e** Quantitative analysis by immunoreactivity of BDNF expression of the PBS-Reg group, BCG-Reg group, PBS-Enr group, and the BCG-Enr group. **f**, **g** Quantitative analyses by RT-PCR of BDNF (**e**) and IGF-1 (**f**) mRNA levels from the BCG-Enr group treated with minocycline or PBS and the BCG-Enr group treated with anti-IL-10 or IgG isotype antibodies. *p < 0.05, **p < 0.01, ***p < 0.001 between the indicated groups by Student’s *t* test (*n* = 6 per group). *Scale bars* 20 μm in **a**–**d**. The data are presented as the means ± SEMs

Astrocytes are localized to the choroid membranes in close spatial proximity to the meningeal areas and the CSF and thus can be modulated by cytokines from both of these areas. We next measured the expression of neurotrophic factors in the third ventricle. A significant increase in BDNF fluorescent staining was observed only in choroid astrocytes from the BCG-Enr mice compared with that in choroid astrocytes of the PBS-Enr mice (Fig. 9a–e; vaccination, *F*_(1, 20)_ = 87.422, *p* < 0.001; Enr, *F*_(1, 20)_ = 130.007, *p* < 0.0001; *n* = 6; two-way ANOVA). Our findings that the BCG-Enr mice showed significantly higher levels of BDNF and IGF-1 expression relative to the PBS-Enr mice support that the third ventricle was linked closely with areas of the hippocampus involved in cognitive function (Fig. 5d, e). Similar phenomenon could be observed when neurotrophic factor expression between the PBS-Reg mice and the BCG-Reg mice was compared (Fig. 5d, e). To link the elevation in neurotrophic factor levels found in the BCG-Enr mice with meningeal macrophage activation, we injected minocycline or vehicle intraperitoneally into the BCG-Enr mice and the PBS-Reg mice. We verified that the increased levels of BDNF and IGF-1 mRNA were significantly blocked after minocycline treatment (Fig. 9f, g; BDNF, *p* < 0.05, IGF-1, *p* < 0.001; *n* = 6). Because many activated macrophages express anti-inflammatory IL-10, we injected neutralizing anti-IL-10 antibodies (BD Bioscience; 60 μg/mouse) and isotype control IgG antibodies into the BCG-Enr mice. The results showed that elevated expression of IGF-1 in the BCG-Enr mice was reversed by anti-IL-10 antibody treatment; however, injection of anti-IL-10 antibodies in the BCG-Enr mice only produced a trend toward decreased BDNF (Fig. 9f; *p* = 0.079), indicating that another anti-inflammatory cytokines, such as IL-4 or TGF-β, may play a role in the activation of meningeal macrophages. In summary, these results suggest that BCG-Enr-induced meningeal macrophage activation contributes to neurotrophic factor BDNF and IGF-1 expression, which is beneficial to hippocampal-dependent cognitive function.

## Discussion

We previously reported that neonatal BCG vaccination elevated neurogenesis, synaptic plasticity, cognitive function, and the levels of neurotrophic factors in mice housed in a standard environment by affecting the neuroimmune cross talk between the periphery and the brain, which may be associated with a systemic Th1 bias [8–10]. Here, we extended the neurobeneficial effects caused by BCG vaccination to the enriched housing condition. We further showed that peripheral immune activation induced by BCG vaccination resulted in some neurobeneficial effects, such as higher levels of BDNF and IGF-1, through skewing meningeal (pia mater and dura mater) macrophages toward an M2 phenotype, and these effects were prevented by minocycline and anti-IL-10 antibody treatment. Importantly, we verified that recruitment of T lymphocytes to the choroid plexus (CP) contributed to meningeal macrophage M2 activation, and these effects could be blocked after anti-TCR antibody treatment. The increased recruitment of T cells to the CP paralleled observed increase in macrophage M2 polarization in the meningeal space. Similarly, the expression of IL-10 (an anti-inflammatory cytokine) in the CP was decreased after anti-TCR antibody treatment, implying that depletion of T cells and change of cytokine IL-10 are coincidence in the CP. Collectively, the present work confirmed that T lymphocytes in the brain ventricle’s CP, meningeal macrophage M2 polarization, microglia M2 activation, and formation of pro-neurogenic niche may combine and contribute to cellular and behavioral differences observed between the BCG-Enr mice and PBS-Enr mice.

The interaction between peripheral immune responses and the CNS has been addressed for decades. However, no plausible immune cellular mechanisms have yet been confirmed to explain the relationship between these two systems. Recently, the “brain border”-meninges including the outermost dura mater, the intermediate arachnoid mater, and the inner pia mater have received increased attention [29]. Notably, the anatomical structures of meninges, such as the choroid membranes/plexus, connect peripheral blood system to the brain via CSF circulation [31, 32]. Therefore, meningeal immunity could be a potential target for elaborating the periphery-to-brain communication pathway. Our findings of elevated recruitment of T lymphocytes into the CP and dura mater and meningeal macrophage M2 polarization in response to BCG vaccination provided evidence for the interior relationship between the periphery and the brain. However, how do the infiltrating T lymphocytes regulate macrophage activation in the meninges? We showed that the level of monocyte-derived anti-inflammatory IL-10, which supports beneficial effects on cognitive function, was elevated in the meninges including pia and dura mater, although IL-10 immunoreactivity was not only restricted to macrophages (Fig. 7h). Accordingly, Jonathan Kipnis’ group [14] also demonstrated that accumulation of another inflammation-resolving cytokine IL-4 was detected in the meninges, in which the involved myeloid cells underwent an inflammatory phenotypic change. In the absence of IL-4, the meningeal myeloid cells were skewed toward a proinflammatory phenotype, which resulted in cognitive impairment in IL-4 knockout mice. Other mechanisms, such as IL-4 and Arg-1, may also play important roles in regulating of macrophage M2 polarization, since increased expression of Arg-1 was observed in the meninges, lateral and third ventricles, and hippocampal CA1 area of the brain (Additional file 3: Figure S4A-F). Choroidal astrocytes are in close proximity to the “brain borders” and the CSF and thus could be mutually regulated by cytokines from the meninges and the CSF. We also showed that astrocytes located in the choroid membrane of the brain ventricle expressed higher levels of BDNF in the BCG-Enr mice than in the PBS-Enr mice. Expectedly, the expression of neurotrophic factors (BDNF and IGF-1) was reversed in the hippocampus by inhibiting meningeal macrophage activation after minocycline or anti-IL-10 antibody treatment. In line with our findings, another anti-inflammatory cytokine IL-4 directly induced BDNF mRNA upregulation in astrocytes in previously published literature [14]. Together with our findings that the levels of IL-10 in the pia and dura mater were also normalized when macrophage activation was inhibited via minocycline treatment, we concluded that meningeal macrophages-derived IL-10 not only directly regulated choroid astrocytes secretion of BDNF but also induced granular neurons expressing BDNF and microglia expressing IGF-1 in the hippocampus after IL-10 release into the CSF. Thus, the findings from us and others revealed that infiltrating T cells in the “brain border” indeed cross talk with resident macrophages secreting the anti-inflammatory cytokine IL-10 and choroidal astrocytes expressing BDNF in addition to cross talk with resident microglia secreting IGF-1 [8, 11]. Notably, the benefits from Enr and even BCG vaccination on neurogenesis may not be exclusively caused by the M2 shift; other mechanisms, such as direct effects from circulatory endothelium-derived factors and MHC-II^+^ cell-derived signals, may also mediate hippocampal neurogenesis [33]. Vascular endothelial growth factor (VEGF) stimulates adult neuronal progenitor cell proliferation in the hippocampus and has neurogenic and angiogenic properties [34, 35]. Since microglia located in the hippocampus are important for the regulation of adult neurogenesis, chemokine CX3CR1 deficient mice perform adult hippocampal neurogenesis deficit and cognitive impairment [36]. Together, microglia-related signals, chemokines, and other growth factors that affect neural progenitor cell proliferation cannot be excluded.

In the present study, similar tendencies were observed in the levels of cytokines in the serum and hippocampus, although each cytokine was expressed to a different extent. Our immunization approach led to an obvious elevation in cytokine IFN-γ level both in the serum and the hippocampus, which is known to induce the upregulation of specific trafficking molecules for recruitment of T lymphocytes into the meninges [30, 37]. However, the levels of the pro-inflammatory cytokines TNF-a, IL-1β, and IL-6, which are associated with cognitive deficits, were decreased in the hippocampus at postnatal day (PD) 21. Indeed, the levels of pro-inflammatory TNF-a and IL-6 were increased during the initial 2 weeks after BCG vaccination and then decreased at PD 21 (Fig. 8c). The initial elevation of cytokines can be induced by peripheral adaptive immune responses. The meningeal macrophage M2 activation at PD 21 may ameliorate the pro-inflammatory immune response in the hippocampus and thereby decrease the levels of TNF-a and IL-6 mRNA. Compared with the PBS-Reg mice, we found significant elevation in hippocampal neurogenesis, LTP, and cognitive function in the BCG-Reg mice and PBS-Enr mice. Although both BCG vaccination and the enriched environment promoted the recruitment of peripheral T lymphocytes, which skewed macrophages toward an anti-inflammatory phenotype, to the CP and meninges, the levels of cytokines in BCG mice and Enr mice were different. This may contribute to the transience of the immune response and sustainability in an enriched environment. Of course, other molecular mechanisms may also explain the differences between these two groups.

In the present study, we have determined infiltrating T cells from periphery to the meninges were significantly increased 2 weeks after BCG vaccination and/or Enr exposure compared to the corresponding controls (Additional file 5: Figure S3A). However, we cannot rule out the possibility of the resident T cell proliferation in the meninges after BCG and/or Enr treatment. Moreover, quantitative analysis revealed no significant changes in CD11b^+^ macrophages in the meninges among all groups, implying that macrophage M2 polarization rather than macrophage migration was affected in the meninges after BCG vaccination (Additional file 5: Figure S3B). Actually, BCG vaccination indeed had a long-lasting effect, since the increases of cytokine levels in the periphery and T cell count in the meninges were still detected 2 weeks after BCG vaccination (Additional file 5: Figure S3D). Together, we agree that resident macrophages were activated, at least in part, by infiltrating T cell-derived cytokines, in the meninges, but not migrate to the meninges from the periphery. Also, homing monocytes were not detected via their in situ labeling by intravenously injected FITC-conjugated anti-CD11b antibodies, further supported our conclusions (Additional file 6: Figure S5C and D).

Recent reports demonstrated that striking Th2 skewing of T lymphocytes potentially led to meningeal myeloid cell M2 polarization [38, 39]. However, our findings regarding the T helper (Th) 1 cytokine-IFN-γ were continuously increased in the periphery and the brain and were specialized to recruit T lymphocytes to the CP and induce macrophage M2 polarization. It seems that our findings of Th1 biased-macrophage M2 activation are contradictory with those of previously reported studies. Notably, a recent study reported that depletion of T cells from meningeal space shewed meningeal myeloid cells toward a proinflammatory phenotype (M1 activation) [14]. Therefore, we formulate a concept that infiltrating T cells and meningeal macrophages maintains a homeostasis under physiological conditions. Alteration of T cell count may modulate meningeal macrophage phenotype. The data revealed that treatment with IFN-γ induced the expression of CCL5, which is important to T cell recruitment. We have also demonstrated that infiltrating T cells to the CP is a requirement for meningeal macrophage M2 activation due to anti-TCR antibody blocking experimental. However, the underlying mechanism remains unclear. We are going to measure which immunomodulatory factors secreted by infiltrating T cells regulating macrophage M2 activation in the further research.

Recently, the brain’s CP activation for recruitment of inflammation-resolving monocytes and T lymphocytes attenuated Alzheimer’s disease (AD) pathology or disease progression in ALS model following glatiramer acetate [40] or myelin-derived peptide [13] immunization, with concomitant Th1-cytokine IFN-γ upregulation in the periphery. Our data now demonstrate that peripheral Th1-meningeal macrophage M2 activation may be an underlying mechanism for interpreting the beneficial effects observed in previous studies [13, 40]. These findings may shed light on the migration of immunoregulatory cells to the CNS for fighting off the neuronal pathology by peripheral immunity.

## Conclusions

In summary, our results show that combined neonatal BCG vaccination and exposure to an enriched environment induced combined neurobeneficial effects and extended the implications of complex neuroimmune communication via a novel peripheral blood-CP-CSF-pia mater-brain-dura mater-CNS lymphatic vessel pathway. Briefly, BCG vaccination and/or enriched environment elicited peripheral Th1 bias and recruitment of T lymphocytes to the CP followed by interactions with macrophages in the meninges and secretion of anti-inflammatory IL-10, which could induce the production of neurotrophic factors (BDNF and IGF-1), and eventually exert beneficial effects on cognitive function.

## Additional files

Additional file 1: Table S1. List of primes used in RT-PCR experiments. **Table S2.** The experimental schedule in the study. (DOC 20 kb)
Additional file 2: Figure S2. Combined effect of BCG vaccination and Enr exposure on neurogenesis in the hippocampal DG. (A) Representative micrographs of the DG stained for BrdU (red). (B) Quantification of BrdU+ cells in the DG of the four groups. **p* < 0.05, two-way ANOVA, followed by LSD post hoc test; *n* = 6 per group. Scale bars: 50 μm. The data are presented as the means ± SEMs. (TIF 994 kb)
Additional file 3: Figure S4. BCG vaccination and/or Enr exposure induced macrophage/microglia expressing Arginase-1 in the meninges and the brain. (A) Representative micrographs of the dura mater stained for Arg-1. (B) A higher magnification (×40) of the inset boxed area in A. (C) Representative micrographs staining for Arg-1 and CD11b in the hippocampus. (D) A higher magnification (×40) of the inset boxed area in C. (E–G) Representative micrographs of the pia mater (E) and lateral (F) and third (G) ventricles stained for CD11b (red) and Arg-1 (green) and with nuclear staining for Hoechst (blue). (H and I) The graphs show expression analyses of the Arg-1 gene and Ym1 gene in the hippocampi of four groups. ***p* < 0.01, ****p* < 0.001 between the indicated groups, two-way ANOVA, followed by LSD post hoc test; *n* = 3 per group. Scale bars: 100 μm in A, C, D and F; 20 μm in B, E and G. The data are presented as the means ± SEMs. (TIF 2655 kb)
Additional file 4: Figure S1. Correlation analysis was assessed via the IL-10 mRNA levels in the dura mater (A) and the pia mater (B) and the number of CD3^+^ T cells in the CP (dura mater: *r* ^2^ = 0.254, *p* < 0.05; pia mater: *r* ^2^ = 0.510, *p* < 0.002). The data are presented as the means ± SEMs. (TIF 114 kb)
Additional file 5: Figure S3. BCG vaccination and/or Enr exposure recruited T cells, but not macrophage to the dura mater form the periphery. (A-B) Representative micrographs of the dura mater stained for CD3e (red), Lyve-1 (green) and Hoechst (blue) in A; stained for CD11b (red) and Hoechst (blue) in B of the four groups. (C) Representative micrographs of the CP stained for MHC-II (green) and Hoechst (blue). (D-F) Quantitative analyses of the number of CD3e^+^ cells (D) in the dura mater, CD11b^+^ cells (E) in the dura mater and MHC-II^+^ cells in the CP (F). **p* < 0.05, ***p* < 0.01, ****p* < 0.001 between the indicated groups, two-way ANOVA, followed by LSD post hoc test; *n* = 6 per group. Scale bars: 100 μm. The data are presented as the means ± SEMs. (TIF 3772 kb)
Additional file 6: Figure S5. Intravenous injection of recombinant IFN-γ recruits T cells to the CP from the periphery. (A-B) Representative micrographs of the dura mater stained for CD3e (red) and Hoechst (blue) following intravenous injection of IFN-γ (1 ng per mice; *n* = 3). (C-D) Homing monocytes were not detected via their in situ labeling by intravenously injected FITC-conjugated anti-CD11b antibodies (Biolegend; 2 μg in 200 μl PBS); representative pictures of whole mounts of excised dura mater are shown (2 weeks after BCG vaccination; *n* = 3). (E) Quantitative analysis of CD3e^+^ T cells in the dura mater in the mice treated with recombinant IFN-γ (1 ng, NeoBioscience) and IgG. (F) The graphs show expression analysis of the CCL-5 gene in the hippocampi of the four groups. **p* < 0.05, ***p* < 0.01 between the indicated groups, student’s *t* test in E; two-way ANOVA, followed by LSD post hoc test in F, *n* = 3 per group. (TIF 1697 kb)

## Abbreviations

BCG: Bacillus Calmette-Guérin
CNS: Central nervous system
CP: Choroid plexus
CSF: Cerebrospinal fluid
Enr: Enriched environment
MWM: Morris water maze
PFA: Paraformaldehyde
PW: Postnatal week
