# Supplementary material for: Combined effect of BCG vaccination and enriched environment promote neurogenesis and spatial cognition via a shift in meningeal macrophage M2 polarization
Source: J Neuroinflammation. 2017 Feb 10;14:32. doi: 10.1186/s12974-017-0808-7 (PMC5301319; doi:10.1186/s12974-017-0808-7)
Supplement: Additional file 1: Table S1. — List of primes used in RT-PCR experiments. Table S2. The experimental schedule in the study. (DOC 20 kb) [file 12974_2017_808_MOESM1_ESM.doc]

**Figure S1.** Correlation analysis was assessed via the IL-10 mRNA levels in the dura mater (A) and the pia mater (B) and the number of CD3^+^ T cells in the CP. (dura mater: *r*^2^ = 0.254, *p* < 0.05; pia mater: *r*^2^ = 0.510, *p* < 0.002). The data are presented as the means ± SEMs.

**Figure S2.** Combined effect of BCG vaccination and Enr exposure on neurogenesis in the hippocampal DG. (A) Representative micrographs of the DG stained for BrdU (red). (B) Quantification of BrdU+ cells in the DG of the four groups. *p < 0.05, two-way ANOVA, followed by LSD post hoc test; n = 6 per group. Scale bars: 50 μm. The data are presented as the means ± SEMs.

**Figure S3.** BCG vaccination and/or Enr exposure recruited T cells, but not macrophage to the dura mater form the periphery. (A-B) Representative micrographs of the dura mater stained for CD3e (red), Lyve-1 (green) and Hoechst (blue) in A; stained for CD11b (red) and Hoechst (blue) in B of the four groups. (C) Representative micrographs of the CP stained for MHC-II (green) and Hoechst (blue). (D-F) Quantitative analyses of the number of CD3e^+^ cells (D) in the dura mater, CD11b^+^ cells (E) in the dura mater and MHC-II^+^ cells in the CP (F). **p* < 0.05, ***p* < 0.01, ****p* < 0.001 between the indicated groups, two-way ANOVA, followed by LSD *post hoc* test; n = 6 per group. Scale bars: 100 μm. The data are presented as the means ± SEMs.

**Figure S4.** BCG vaccination and/or Enr exposure induced macrophage/microglia expressing Arginase-1 in the meninges and the brain. (A) Representative micrographs of the dura mater stained for Arg-1. (B) A higher magnification (40×) of the inset boxed area in (A). (C) Representative micrographs staining for Arg-1 and CD11b in the hippocampus. (D) A higher magnification (40×) of the inset boxed area in (C). (E-G) Representative micrographs of the pia mater (E) and lateral (F) and 3rd (G) ventricles stained for CD11b (red), Arg-1(green) and with nuclear staining for Hoechst (blue). (H and I) The graphs show expression analyses of the Arg-1 gene and Ym1 gene in the hippocampi of four groups. ***p* < 0.01, ****p* < 0.001 between the indicated groups, two-way ANOVA, followed by LSD *post hoc* test; n = 3 per group. Scale bars: 100 μm in A, C, D and F, 20 μm in B, E and G. The data are presented as the means ± SEMs.

**Figure S5.** Intravenous injection of recombinant IFN-γ recruits T cells to the CP from the periphery. (A-B) Representative micrographs of the dura mater stained for CD3e (red) and Hoechst (blue) following intravenous injection of IFN-γ (1 ng per mice; n = 3). (C-D) Homing monocytes were not detected via their in-situ labeling by intravenously injected FITC-conjugated anti-CD11b antibodies (Biolegend; 2 µg in 200µl PBS); representative pictures of whole mounts of excised dura mater are shown (2 week after BCG vaccination; n = 3). (E) Quantitative analysis of CD3e^+^ T cells in the dura mater in the mice treated with recombinant IFN-γ (1 ng, NeoBioscience) and IgG. (F) The graphs show expression analysis of the CCL-5 gene in the hippocampi of the four groups. **p* < 0.05, ***p* < 0.01 between the indicated groups, student’s t test in E; two-way ANOVA, followed by LSD *post hoc* test in F, n = 3 per group.

**Table S1. List of primes used in RT -PCR experiments.**

| Gene name | Sequence |
| --- | --- |
| IFN-γ  IL-1β | Forward 5’-AGCGGCTGACTGAACTCAGATTGTAG-3’  Reverse 5’-GTCACAGTTTTCAGCTGTATAGGG-3’  Forward 5’-TGTCTTTCCCGTGGACCTTC-3’ |
|  | Reverse 5’-CTAATGGGAACGTCACACACC-3’ |
| IL-6  TNF-a  IL-10  VCAM-1  ICAM-1  BDNF  IGF-1  Arginase-1  Ym-1  CCL5  β-actin | Forward 5’-TCTGGGAAATCGGGAAATGAG-3’  Reverse 5’-TCTCTGAAGGACTCTGGCTTTGTC-3’  Forward 5’-ACAAGGCTGCCCCGACTAT-3’  Reverse 5’-CTCCTGGTATGAAGTGGCAAATC-3’  Forward 5’- TAAGGGTTACCTGGGTTGCCAAG -3’  Reverse 5’- CAAATGCTCCTTGATTTCTGGGC -3’  Forward 5’-TGTGAAGGGATTAACGAGGCTGGA-3’  Reverse 5’-CCATGTTTCGGGCACATTTCCACA-3’  Forward 5’-AGATCACATTCACGGTGCTGGCTA-3’  Reverse 5’-AGCTTTGGGATGGTAGCTGGAAGA-3’  Forward 5’-GAGGGCTCCTGCTTCTCAA-3’  Reverse 5’- GCCTTCATGCAACCGAAGT -3’  Forward 5’- AAGGCAGTTTACCCAGGCTC -3’  Reverse 5’- TCTTTATTGCAGGTGCGGTCA -3’  Forward 5’-AGCCAATGAAGAGCTGGCTGGT-3’  Reverse 5’-AACTGCCAGACTGTGGTCTCCA-3’  Forward 5’-GGCATACCTTTATCCTGAG-3’  Reverse 5’-CCACTGAAGTCATCCATGTC-3’  Forward 5’-GTGCTCCAATCTTGCAGTCGTGTT-3’  Reverse 5’-ACTTCTTCTCTGGGTTGGCACACA-3’  Forward 5’-GGTACCACCATGTACCCAGG-3’  Reverse 5’-ACATCTGCTGGAAGGTGGAC-3 |

**Table S2. The experimental schedule in the study**

| Experiment | Week of Treatment | Times | Approaches |
| --- | --- | --- | --- |
| Anti-TCR Abs injection  Minocycline injection  IFN-γ injection  Anti-IL-10 Abs | PW3  PW3 to PW4  PW3 to PW4  PW3 | 1  7  7  1 | i.c.v.  i.p.  i.c.v  i.c.v. |
