# Supplementary figures and images for: Combined effect of BCG vaccination and enriched environment promote neurogenesis and spatial cognition via a shift in meningeal macrophage M2 polarization
Source: J Neuroinflammation. 2017 Feb 10;14:32. doi: 10.1186/s12974-017-0808-7 (PMC5301319; doi:10.1186/s12974-017-0808-7)

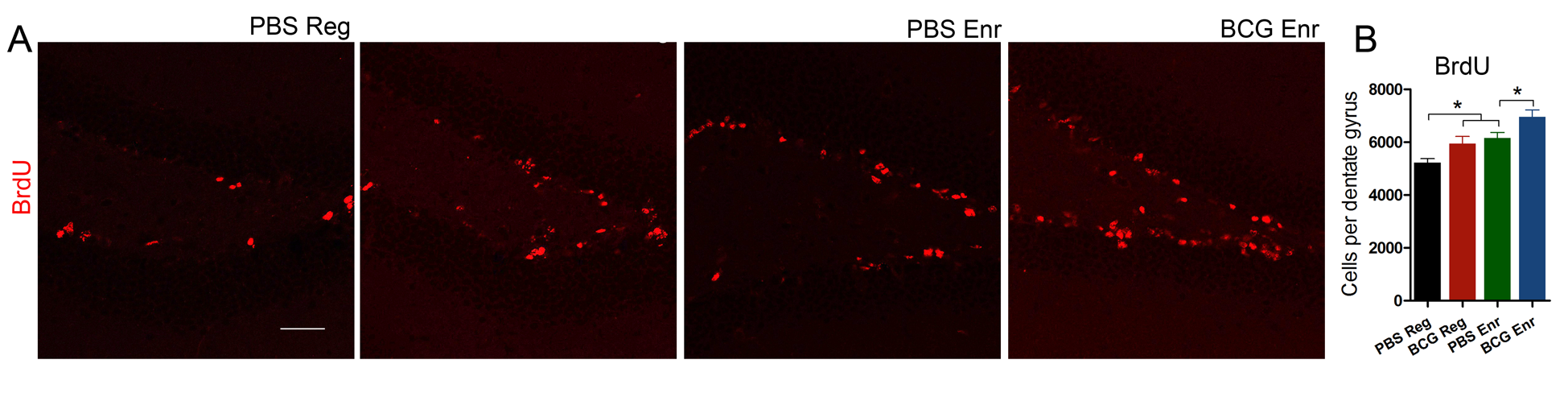

Supplement: Additional file 2: Figure S2. — Combined effect of BCG vaccination and Enr exposure on neurogenesis in the hippocampal DG. (A) Representative micrographs of the DG stained for BrdU (red). (B) Quantification of BrdU+ cells in the DG of the four groups. *p < 0.05, two-way ANOVA, followed by LSD post hoc test; n = 6 per group. Scale bars: 50 μm. The data are presented as the means ± SEMs. (TIF 994 kb) [file 12974_2017_808_MOESM2_ESM.tif]

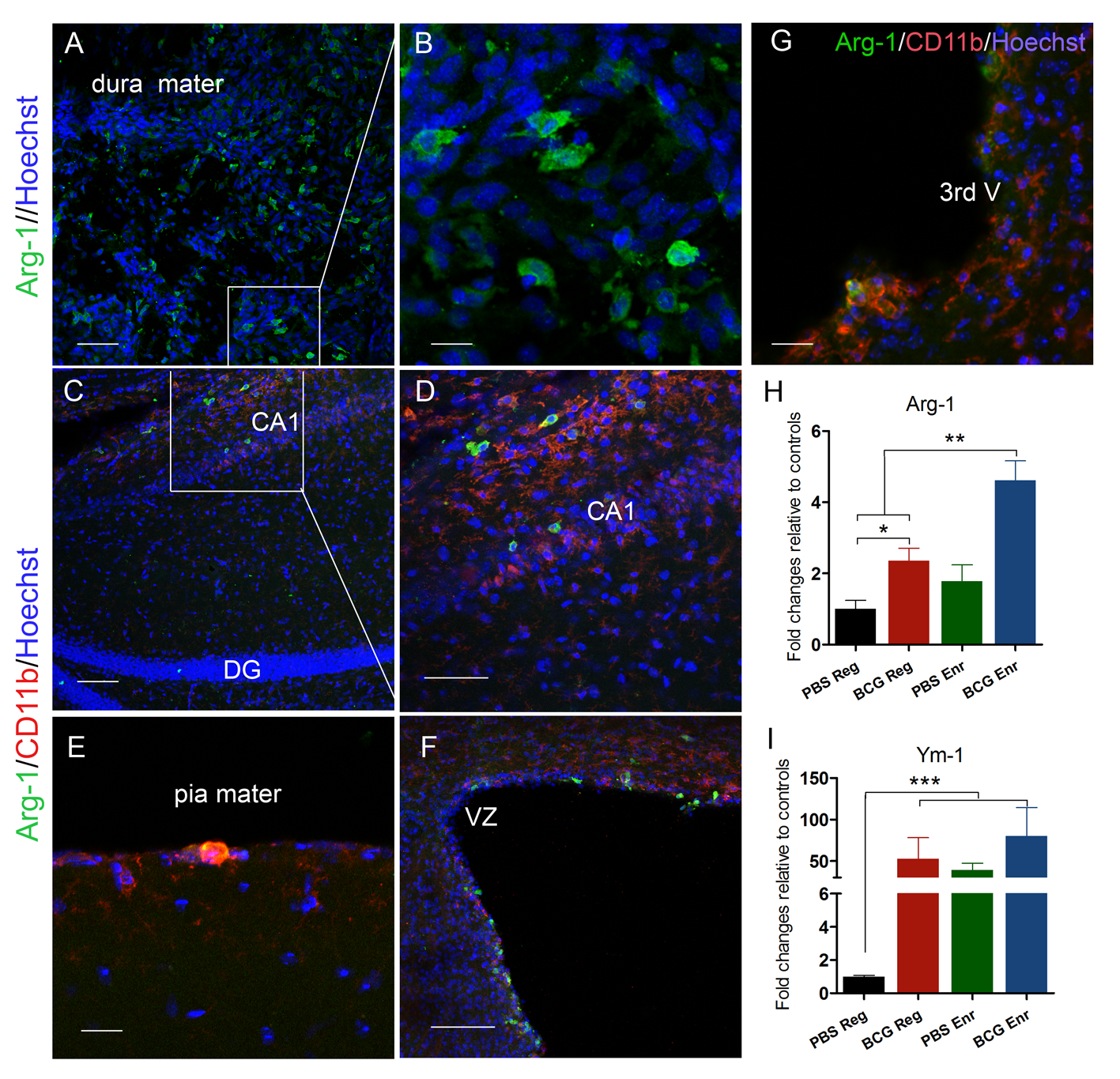

Supplement: Additional file 3: Figure S4. — BCG vaccination and/or Enr exposure induced macrophage/microglia expressing Arginase-1 in the meninges and the brain. (A) Representative micrographs of the dura mater stained for Arg-1. (B) A higher magnification (×40) of the inset boxed area in A. (C) Representative micrographs staining for Arg-1 and CD11b in the hippocampus. (D) A higher magnification (×40) of the inset boxed area in C. (E–G) Representative micrographs of the pia mater (E) and lateral (F) and third (G) ventricles stained for CD11b (red) and Arg-1 (green) and with nuclear staining for Hoechst (blue). (H and I) The graphs show expression analyses of the Arg-1 gene and Ym1 gene in the hippocampi of four groups. **p < 0.01, ***p < 0.001 between the indicated groups, two-way ANOVA, followed by LSD post hoc test; n = 3 per group. Scale bars: 100 μm in A, C, D and F; 20 μm in B, E and G. The data are presented as the means ± SEMs. (TIF 2655 kb) [file 12974_2017_808_MOESM3_ESM.tif]

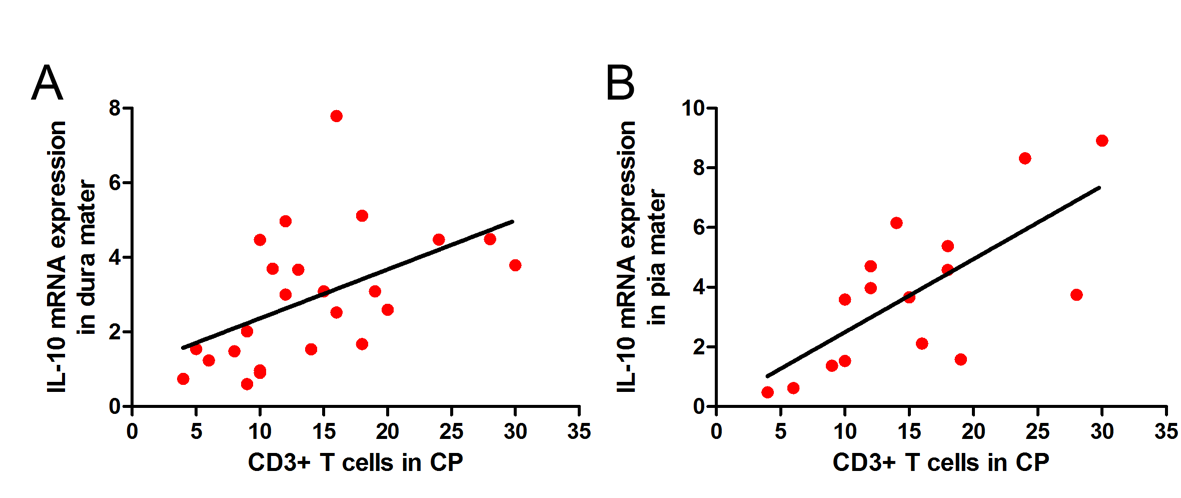

Supplement: Additional file 4: Figure S1. — Correlation analysis was assessed via the IL-10 mRNA levels in the dura mater (A) and the pia mater (B) and the number of CD3+ T cells in the CP (dura mater: r 2 = 0.254, p < 0.05; pia mater: r 2 = 0.510, p < 0.002). The data are presented as the means ± SEMs. (TIF 114 kb) [file 12974_2017_808_MOESM4_ESM.tif]

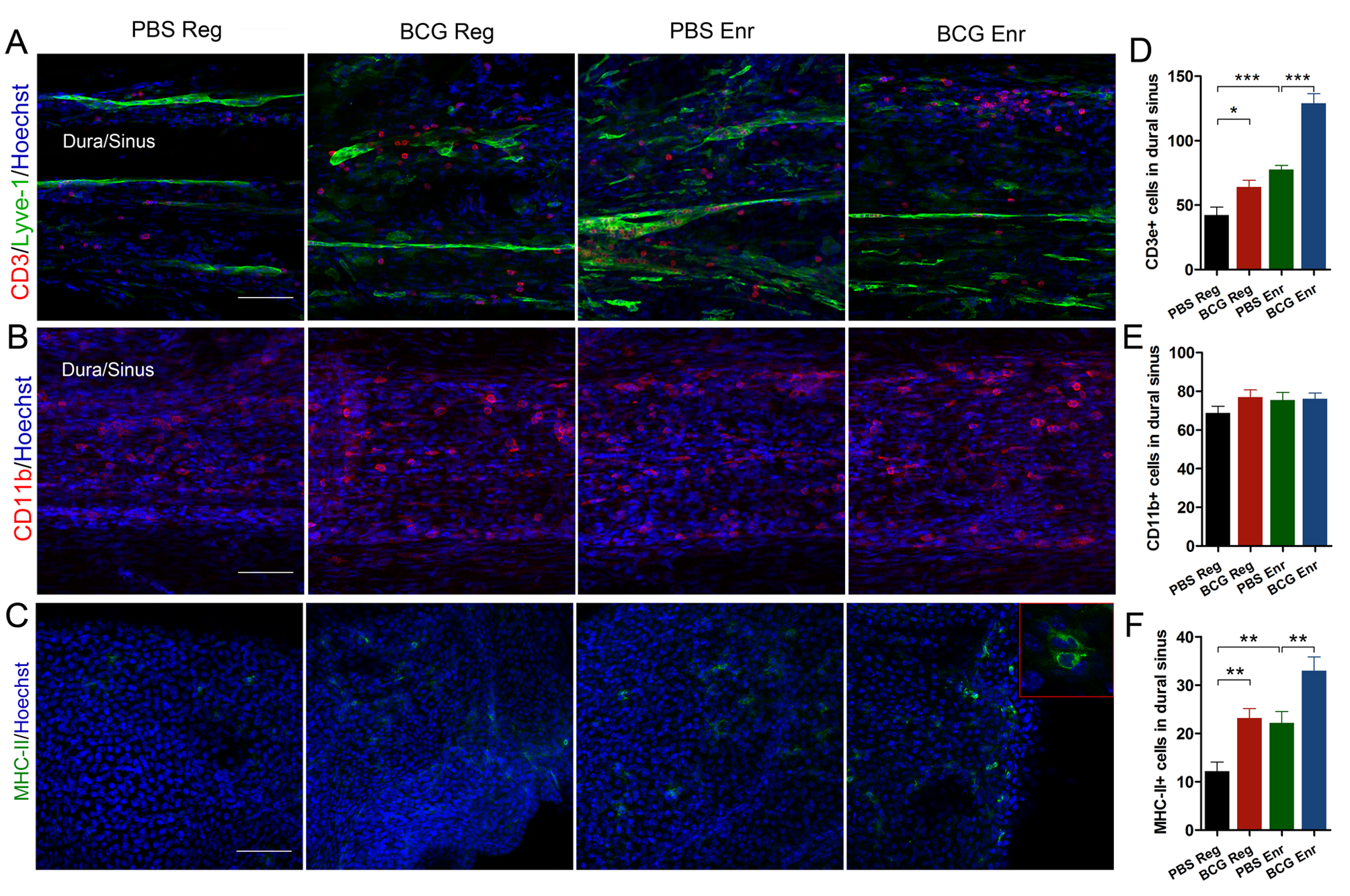

Supplement: Additional file 5: Figure S3. — BCG vaccination and/or Enr exposure recruited T cells, but not macrophage to the dura mater form the periphery. (A-B) Representative micrographs of the dura mater stained for CD3e (red), Lyve-1 (green) and Hoechst (blue) in A; stained for CD11b (red) and Hoechst (blue) in B of the four groups. (C) Representative micrographs of the CP stained for MHC-II (green) and Hoechst (blue). (D-F) Quantitative analyses of the number of CD3e+ cells (D) in the dura mater, CD11b+ cells (E) in the dura mater and MHC-II+ cells in the CP (F). *p < 0.05, **p < 0.01, ***p < 0.001 between the indicated groups, two-way ANOVA, followed by LSD post hoc test; n = 6 per group. Scale bars: 100 μm. The data are presented as the means ± SEMs. (TIF 3772 kb) [file 12974_2017_808_MOESM5_ESM.tif]

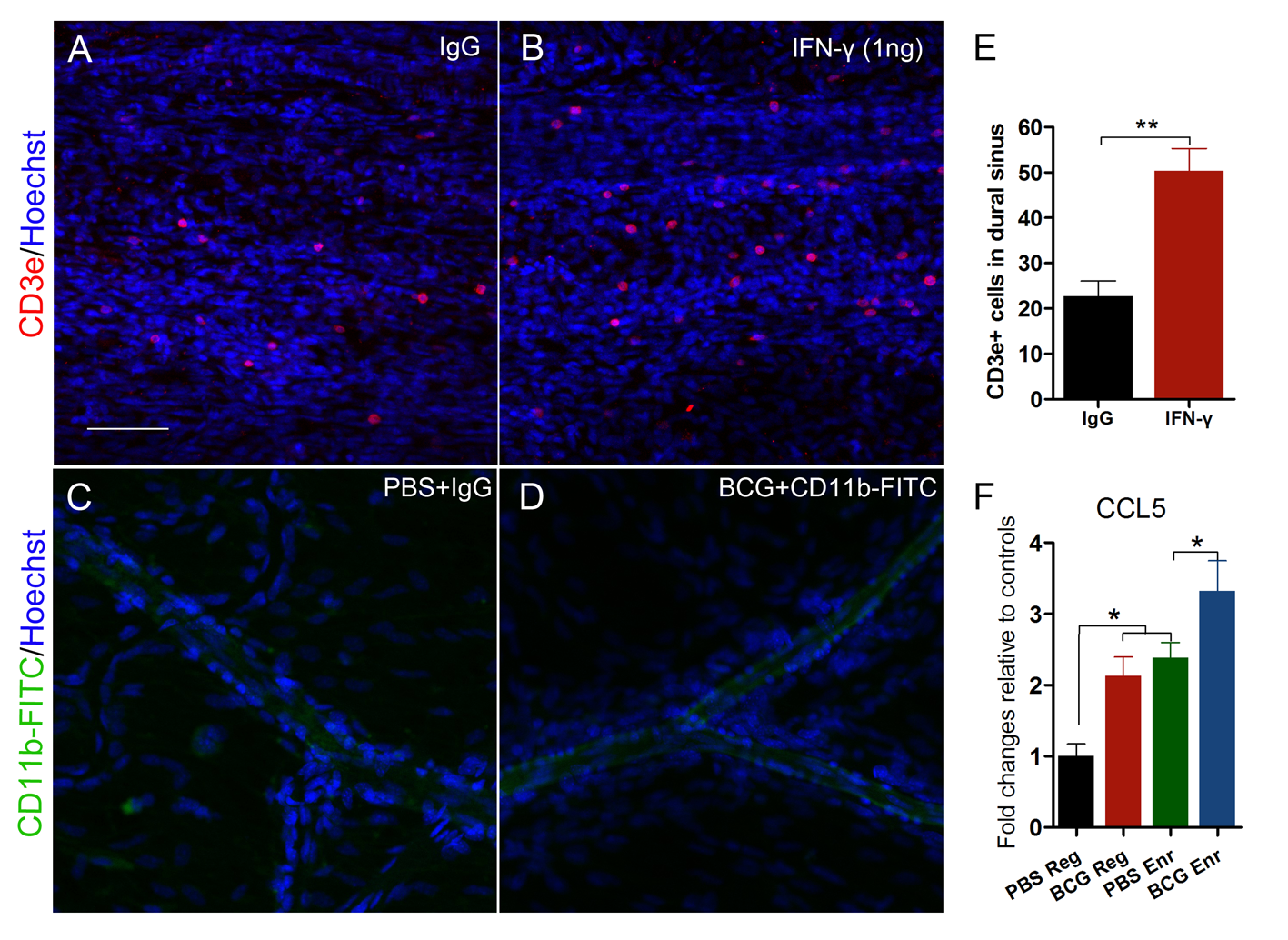

Supplement: Additional file 6: Figure S5. — Intravenous injection of recombinant IFN-γ recruits T cells to the CP from the periphery. (A-B) Representative micrographs of the dura mater stained for CD3e (red) and Hoechst (blue) following intravenous injection of IFN-γ (1 ng per mice; n = 3). (C-D) Homing monocytes were not detected via their in situ labeling by intravenously injected FITC-conjugated anti-CD11b antibodies (Biolegend; 2 μg in 200 μl PBS); representative pictures of whole mounts of excised dura mater are shown (2 weeks after BCG vaccination; n = 3). (E) Quantitative analysis of CD3e+ T cells in the dura mater in the mice treated with recombinant IFN-γ (1 ng, NeoBioscience) and IgG. (F) The graphs show expression analysis of the CCL-5 gene in the hippocampi of the four groups. *p < 0.05, **p < 0.01 between the indicated groups, student’s t test in E; two-way ANOVA, followed by LSD post hoc test in F, n = 3 per group. (TIF 1697 kb) [file 12974_2017_808_MOESM6_ESM.tif]
